# Targeting the smooth muscle cell Keap1-Nrf2-GSDMD-pyroptosis axis by cryptotanshinone prevents abdominal aortic aneurysm formation

**DOI:** 10.7150/thno.98400

**Published:** 2024-10-07

**Authors:** Jiaojiao Wang, Weile Ye, Jiami Zou, Pinglian Yang, Mei Jin, Zhihua Zheng, Chunhong Zhou, Wanlu Qiu, Jing Lu, Chengzhi Li, Shuai Guo, Yiming Xu, Zunnan Huang, Peiqing Liu, Zhiping Liu

**Affiliations:** 1 Key Laboratory of Big Data Mining and Precision Drug Design of Guangdong Medical University, Key Laboratory of Computer-Aided Drug Design of Dongguan City, Key Laboratory for Research and Development of Natural Drugs of Guangdong Province, School of Pharmacy, Guangdong Medical University, Dongguan, 523808, China.; 2 State Key Laboratory of Bioactive Molecules and Druggability Assessment, Jinan University, Guangzhou, 511443, China.; 3 Guangdong Province Key Laboratory of Pharmacodynamic Constituents of Traditional Chinese Medicine and New Drugs Research, College of Pharmacy, Jinan University, Guangzhou, 511443, China.; 4 National and Local United Engineering Lab of Druggability and New Drugs Evaluation, School of Pharmaceutical Sciences, Sun Yat-sen University, Guangzhou, 510006, China.; 5 Guangdong Provincial Key Laboratory of New Drug Design and Evaluation, Guangdong Province Engineering Laboratory for Druggability and New Drug Evaluation, School of Pharmaceutical Sciences, Sun Yat-sen University, Guangzhou, 510006, China.; 6 Department of Interventional Radiology and Vascular Surgery, The First Affiliated Hospital of Jinan University, Guangzhou, 510632, China.; 7 School of Basic Medical Sciences, State Key Lab of Respiratory Disease, Guangzhou Medical University, Guangzhou, 511436, China.

## Abstract

**Rationale**: Abdominal aortic aneurysm (AAA) is an inflammatory, fatal aortic disease that currently lacks any effective drugs. Cryptotanshinone (CTS) is a prominent and inexpensive bioactive substance derived from *Salvia miltiorrhiza Bunge*, a well-known medicinal herb for treating cardiovascular diseases through its potent anti-inflammatory properties. Nevertheless, the therapeutic effect of CTS on AAA formation remains unknown.

**Methods**: To investigate the therapeutic effect of CTS in AAA, variety of experimental approaches were employed, majorly including AAA mouse model establishment, real-time polymerase chain reaction (PCR), RNA sequencing, western blot, co-immunoprecipitation, scanning/transmission electron microscopy (SEM/TEM), enzyme-linked immunosorbent assay (ELISA), seahorse analysis, immunohistochemistry, and confocal imaging.

**Results**: In this study, we demonstrated that CTS suppressed the formation of AAA in apolipoprotein E knock-out (ApoE^-/-^) mice infused with Ang II. A combination of network pharmacology and whole transcriptome sequencing analysis indicated that activation of the Keap1-Nrf2 pathway and regulation of programmed cell death in vascular smooth muscle cells (VSMCs) are closely linked to the anti-AAA effect of CTS. Mechanistically, CTS promoted the transcription of Nrf2 target genes, particularly Hmox-1, which prevented the activation of NLRP3 and GSDMD-initiated pyroptosis in VSMCs, thereby mitigating VSMC inflammation and maintaining the VSMC contractile phenotype. Subsequently, by utilizing molecular docking, together with the cellular thermal shift assay (CETSA) and isothermal titration calorimetry (ITC), a particular binding site was established between CTS and Keap1 at Arg415. To confirm the binding site, site-directed mutagenesis was performed, which intriguingly showed that the Arg415 mutation eliminated the binding between CTS and the Keap1-Nrf2 protein and abrogated the antioxidant and anti-pyroptosis effects of CTS. Furthermore, VSMC-specific Nrf2 knockdown in mice dramatically reversed the protective action of CTS in AAA and the inhibitory effect of CTS on VSMC pyroptosis.

**Conclusion**: Naturally derived CTS exhibits promising efficacy as a treatment drug for AAA through its targeting of the Keap1-Nrf2-GSDMD-pyroptosis axis in VSMCs.

## Introduction

Abdominal aortic aneurysm (AAA) is a fatal condition characterized by localized enlargements of the abdominal aorta that predisposes to a clinical emergency of rupture with a rapid death rate exceeding 65% 1, 2. To date, open surgical and endovascular stent graft repairs are the mainstays of AAA treatment, while no pharmacological treatment is available for AAA 3. Therefore, there is great urgency in comprehending its pathogenesis, further identifying novel treatment targets, and developing drug interventions.

The abdominal aorta is classified as an elastic artery, and the resilience and elasticity of the aortic wall are primarily determined by the extracellular matrix (ECM), mainly elastin and fibrillar collagen. The synthesis, secretion, and deposition of elastin are predominantly in the vascular smooth muscle cells (VSMCs), which constitute approximately 70% of the total cell population in the aorta wall 4, 5. As the predominant cell type within the aortic wall, VSMC dysfunction, including inflammation, oxidative stress, and phenotypic switching, has been demonstrated to contribute to AAA formation 1, 2. In addition, rarefaction and depletion of VSMCs via various forms of cell death also characterize AAA 6-9. VSMC-specific-deleting Gasdermin D (GSDMD), a fundamental executive pore-forming protein in pyroptosis, which is a form of programmed cell death tightly linked to inflammation, alleviates Angiotensin II (Ang II)-induced AAA in apolipoprotein E knock-out (ApoE^-/-^) mice 10-12. Meanwhile, chronic inflammation resulting from the infiltration of immune cells into the damaged aortic wall is also a pivotal driver of AAA, which also comprises AAA as an inflammatory disease 13, 14. Elevated levels of tumor necrosis factor α (TNF-α) and interleukin 1 beta (IL-1β) are evident in AAA 15, 16. Cell pyroptosis and inflammation activation form a vicious circle and drive the progression of AAA 12. Thus, strategies targeting cell pyroptosis and inflammation may be effective in treating AAA.

Cryptotanshinone (CTS; PubChem CID: 160254; Molecular Formula: C_19_H_20_O_3_) is a major lipophilic active ingredient derived from the plant *Salvia miltiorrhiza Bunge,* a well-known medicinal herb that has been traditionally used in Asia for treating a range of ailments, particularly cardiovascular and cerebrovascular diseases. We have previously demonstrated that CTS protects against pulmonary fibrosis by blocking the Smad2/3 and STAT3 signaling pathways 17. Additionally, in ApoE^-/-^ mice, CTS significantly reduced the formation of atherosclerotic plaque and improved plaque stability by reducing lectin-like ox-LDL receptor-1 (LOX-1) expression and suppressing NF-κB activation in endothelial cells (ECs) 18. Although CTS has shown remarkable biological and pharmacological properties (e.g., anti-inflammatory, antioxidant, and anti-fibrotic) and cardiovascular protective effects (e.g., anti-atherosclerosis), the potential function and underlying molecular mechanisms of CTS in AAA remain to be investigated.

Natural products that have been meticulously designed and synthesized to aid in evolution are currently acquiring significance in drug discovery 19. In this study, we uncover a protective effect of CTS via the Keap1-Nrf2-GSDMD-pyroptosis axis in AAA utilizing an Ang II-infused ApoE^-/-^ mouse model *in vivo* and a TNF-α-driven cell model *in vitro*. Importantly, the protective effect of CTS is abrogated by the adeno-associated viruses (AAV)-based VSMC-specific Nrf2 knockdown or Nrf2 specific inhibitor ML385, respectively. Furthermore, the specific binding site (Arg415) between CTS and Keap1 has been identified, and site-directed mutagenesis of the Arg415 residue subsequently prevents the binding. Overall, our findings suggest that CTS prevents AAA formation by inhibiting VSMC pyroptosis via competitive binding to Keap1 at the Arg415 residue.

## Materials and Methods

### Animal protocol

All animal experiments were performed in compliance with the Laboratory Animal Ethics Committee of Jinan University (Guangzhou; Approval No.: IACUC-20230625-13). The ApoE^-/-^ mice were randomly assigned to four groups, as described below: 1 Saline group (Saline); 2 Angiotensin II group (Ang II, 1000 ng/kg/min, ALX-151-039, Enzo Life Sciences, NY, USA); 3 Low-dose CTS (S31670, Yuanye Biotechnology, Shanghai, China) treatment group (CTS-L); and 4 High-dose CTS treatment group (CTS-H), n = 15 per group. The murine AAA model was duplicated following established procedures as we previously described 20. For all Angiotensin II infusion procedures, 12-week-old male ApoE^-/-^ (Strain NO. T001458, GemPharmatech, Guangdong, China) mice were anesthetized by isoflurane inhalation (induction concentration: 3-4%, maintenance concentration: 1-2%) and mini-osmotic pumps (0.25 µL/h, 28 consecutive days, Alzet Model 2004, Durect Corporation, CA, USA) containing Ang II were implanted subcutaneously into the dorsum of mice.

During the AAA model setup, the mice were orally administered CTS, which was included in hydroxypropyl-beta-cyclodextrin (HP-β-CD, HY-101103, MedChemExpress, NJ, USA) to enhance the bioavailability of CTS 18, 21, through gavage at either a low dose (15 mg/kg, ~1.22 mg/kg in the human equivalent dose) or a high dose (50 mg/kg, ~4.05 mg/kg in the human equivalent dose) 22 daily for 28 consecutive days. The inclusion complex of CTS using HP-β-CD was prepared as described before 18, 21. Mouse systolic blood pressure was measured at the day before Day 0 and Day 28 following the Ang II infusion using a noninvasive tail-cuff blood pressure measuring device (BP-2000, Visitech Systems, NC, USA). Following the 28-consecutive-day treatment, the mice were euthanized, and the aortas and other organs were then collected for analysis. The greatest diameter of the abdominal aorta was assessed using a digital caliper in a double-blind manner at the endpoint of the experiment. An aortic dilatation above 50% of the standard aorta size is categorized as an aneurysm. The occurrence of a ruptured aorta was considered while examining the frequency of aneurysms, but not when examining the size of the aorta. The assignment of interventions and assessment of experimental outcomes were concealed from the researchers.

### Adeno-associated virus serotype 2 (AAV2)-based VSMC-specific knockdown of Nrf2

To achieve the VSMC-specific knockdown of *Nfe2l2* (gene name of Nrf2), small short hairpin RNA targeted *Nfe2l2* (shRNA, sequence: CTTGAAGTCTTCAGCATGTTA) was cloned and packaged into an AAV2 vector with the VSMC-specific SM22α promoter, which was designed and generated by WZ Biosciences Inc. (Shandong, China). The AAV2 serotype shows the highest transduction efficiency of VSMCs compared to other known serotypes 23. In brief, 10-week-old male ApoE^-/-^ mice were injected through the tail vein with 100 μL (total viral load per mouse: 5.0×10^11^ vg) of AAV2-shRNA-*Nfe2l2* or AAV2-shRNA-Ctrl virus suspension diluted with saline. Two weeks following the virus injection, the murine AAA model was established as mentioned above, and the mice were then administered a high dose of CTS (50 mg/kg).

### Primary culture of rodent aortic VSMCs

Rat aortic VSMCs (RAVSMCs) were isolated and cultured from the rat abdominal aorta. 8-week-old male Sprague-Dawley (SD) rats were obtained from the Guangdong Medical Laboratory Animal Center (Guangdong, China). The rat aorta was carefully removed and placed in pre-cold sterile PBS and the adventitia was then removed completely. Carefully cut open the vessel to expose the intima and gently scrape the intima with curved forceps to remove the endothelial layer. Then, cut the aorta, which only has the smooth muscle layer, into small pieces and put the tissue pieces in a standing T-25 culture flask with 1-2 mL of medium for 2-4 h. During the subsequent cell culture, change the medium every 2-3 days until the cells climb out from the edge of the tissue block and become confluent. The confluent cells were digested with trypsin and planted in a new culture flask for further experiments.

Mouse aortic VSMCs (MAVSMCs) were isolated and cultured as we previously described 20, 27. Briefly, the aortas were excised and immersed in Hank's balanced salt solution (HBSS) with 1 mg/mL collagenase II, 0.744 units/mL elastase, and 1 mg/mL soybean trypsin inhibitor, and incubated at 37 °C for 8 min. Adventitia and endothelium were removed, and the medial layers were further digested with the enzyme solution for 60 min under the same conditions. Cells were cultured in DMEM (CR-12800, Cienry, Zhejiang, China) with the addition of 10% fetal bovine serum (FBS, AB-FBS-1050S, Mogengel Bio, Xiamen, China). RAVSMCs and MAVSMCs were identified by immunofluorescence using the α-SMA antibody. The number of passages used for the cells is between 3 and 6.

### RNA sequencing (RNA-seq)

Total RNA was collected and extracted from different treatment groups in RAVSMCs. RNA quality was assessed by the Agilent 2100 Bioanalyzer (Agilent Technologies, CA, USA) and agarose gel electrophoresis. Then, the mRNA was enriched, fragmented, and transcribed into cDNA and sequenced using Illumina Novaseq 6000 (Gene Denovo Biotechnology Co., Guangdong, China). Differentially expressed genes (DEGs), GO enrichment analysis, and pathway enrichment analysis were performed based on the reads.

### Network pharmacology

A thorough search was conducted on the OMIM (https://omim.org/), Digenet (https://www.disgenet.org/), and Genecards (https://www.genecards.org/) databases to identify a total of 3,694 Homo sapiens targets linked with AAA; 2,537 distinct targets were left after removing duplicates. The TCMSP (http://tcmspnw.com/), SwissTargetPrediction (http://swisstargetprediction.ch/), Pharmmapper (http://www.lilab-ecust.cn/pharmmapper/), and SuperPred (https://prediction.charite.de/) databases were sequentially searched for CTS-related targets, and 439 targets were obtained after removing duplicates. Two target networks were generated for AAA and CTS using Cytoscape 3.10.0 software and its stringApp. Key nodes with degree values exceeding the average were identified by analyzing the intersection nodes of two networks. The OmicShare Platform performed enrichment analysis on the major nodes of AAA and CTS, including Disease Ontology (DO), Kyoto Encyclopedia of Genes and Genomes (KEGG), and Gene Ontology (GO). The data was visually represented using bar charts or bubble charts.

### Scanning electron microscope (SEM)

RAVSMCs were cultured on 10 mm glass coverslips and fixed for 1 h in commercial fixative for SEM (G1102, Servicebio, Hubei, China). The fixed RAVSMCs were washed three times in PBS, post-fixed for 30 min in 1% OsO_4_, dehydrated in an ethanol series, critical-point dried, sputter coated with a thin layer of gold to increase conductivity, and viewed and photographed under SEM (SU-100, Hitachi, Tokyo, Japan).

### Transmission electron microscope (TEM)

The RAVSMCs were pretreated with CTS for 3 h, followed by the addition of TNF-α (10 ng/mL) with an incubation of 24 h. Following treatment, the RAVSMCs were collected and fixed in 2.5% glutaraldehyde overnight in the dark at 4 °C. Subsequently, they underwent further fixation in 1% osmium tetroxide for a duration of 1 h. The fixed specimens were then dehydrated using a succession of ethanol solutions, gradually increasing in concentration until reaching 100% and embedded. Ultrathin sections (60 nm) were sliced, positioned on copper grids, and treated with uranyl acetate and lead citrate before being analyzed using a transmission electron microscope (JEM-1400 Flash, Tokio, Japan) at an accelerating voltage of 80 kV.

### Measurement of mitochondrial function and intracellular redox state

**Intracellular hydrogen peroxide (H_2_O_2_) detection**: Intracellular H_2_O_2_ production was detected based on a previous study 24. Briefly, Amplex-Red (100 μM, ST010, Beyotime, Shanghai, China) and horseradish peroxidase (HRP, 0.25 U/mL) solutions were firstly prepared. Following the indicated treatment, 5×10^6^ cells were trypsinized and permeabilized using a buffer containing 0.001% digitonin dissolved in a 4 mL solution (consisting of 65 mM KCl, 2 mM phosphate, 2 mM EGTA, 125 mM sucrose, 10 mM HEPES, 0.2% BSA, and 2 mM MgCl_2_) at pH 7.2. The reaction was started by adding 50 μL of the Amplex-Red/HRP solution to each well of the microplate, followed by a 30-min incubation at 37 °C in the absence of light. Fluorescence intensity was measured at excitation and emission wavelengths of 563 and 587 nm, respectively. Two different substrate combinations were utilized: one had a mixture of 1 mM malate, glutamate, pyruvate, and succinate; the other contained only 1 mM succinate. The release of peroxide in different stages was regulated by adding specific chemicals: 1 mM ADP (NP0050, Leagene, Beijing, China), 1.3 μM oligomycin (O815255, Macklin, Shanghai, China), 10 μM S1QEL1.1 (GC44860, GlpBio, CA, USA), or 1 μM rotenone (R817233, Macklin, Shanghai, China). Calibration was conducted using a standard curve generated by hydrogen peroxide.

**ROS measurement**: Frozen aortic tissue slices and cultured RAVSMCs were incubated with 10 μM dihydroethidium probe (DHE, D1008, UElandy, Jiangsu, China) for 30 min at 37 °C as we previously described 25, and a fluorescence microscope was applied to take pictures. For ROS-positive cell counting, the DHE probe-incubated cells were digested and re-suspended in cold PBS. A flow cytometer (BD FACSCanto, NJ, USA) was used to count, and FlowJo software (Becton, NJ, USA) was used to analyze the data.

**Superoxide dismutase (SOD) and malondialdehyde (MDA) detection:** SOD and MDA levels in treated RAVSMCs and Ang II-induced murine plasma were measured using the Total Superoxide Dismutase Assay Kit with NBT (S0109, Beyotime, Shanghai, China) and the Lipid Peroxidation MDA Assay Kit (S0131S, Beyotime, Shanghai, China) according to the manufacturer's protocols.

### Expression and purification of His-labeled Keap1 WT and R415K proteins

The *Keap1* gene was amplified with the KOD-Neo-Plus kit (KOD-401, TOYOBO, Osaka, Japan) and subsequently extracted and purified using the QIAquick Gel Extraction Kit (28704, QIAGEN, MD, USA). The pET-28a plasmid harboring the Keap1 gene was double-digested with NdeⅠ and BamHⅠ enzymes (ER0582, ER0051, Thermo Scientific, MA, USA) and then ligated with T4 ligase to add His-tag. The Keap1 R415K plasmid was generated using the Fast Mutagenesis kit (FM111-02, TransGen Biotech, Beijing, China), with the Keap1-WT plasmid serving as a template. The mutation primers are listed in Table S2. Plasmids containing the His-tagged Keap1-WT and Keap1-R415K sequences were transfected into the E. coli BL21 (DE3) strain (CD601-01, TransGen Biotech, Beijing, China), respectively. Then the protein expression was stimulated by the addition of 500 nM IPTG, incubated at 25 °C for 16 h, and isolated using Ni-NTA His-Tag Purification Agarose (HY-K0210, MedChemExpress, NJ, USA).

### Molecular docking

The structure of CTS was determined using the PubChem database (https://pubchem.ncbi.nlm.nih.gov/). The crystal structure of the Keap1 protein was acquired from the RCSB PDB database (https://www.rcsb.org/). The software Molecular Operating Environment 2015 (MOE 2015, Chemical Computing Group ULC, Montreal, Canada) was utilized to construct a molecular docking model of CTS with Keap1.

### Cellular thermal shift assay (CETSA)

RAVSMCs were treated with 10 µM CTS or DMSO for 4 h. Then the cells were washed and harvested in PBS containing protease inhibitors (A32953, Thermo Scientific, MA, USA). The cell mixture was equally divided into eight portions and heated for 5 min at the indicated temperatures. Heated samples stayed at room temperature for 3 min before snap freezing with liquid nitrogen. After two freeze-thaw cycles and centrifugation at 12,000 g for 20 min, the supernatants collected were ready for western blot analysis. For the ITDRF (isothermal dose-response fingerprints)-CETSA assay 26, CTS was exposed to RAVSMCs in escalating concentrations ranging from 0 to 102.4 µM for a duration of 4 h. Cells were collected, and target proteins were detected, as aforementioned.

### Isothermal titration calorimetry (ITC)

The ITC assay was performed using a MicroCal PEAQ-ITC apparatus (Malvern Panalytical, PA, USA). Prior to commencing the experiment, the proteins underwent dialysis in a solution comprising 20 mM Tris-HCl (pH 7.5) and 200 mM NaCl. The CTS powder was dissolved in the same solution with 6% DMSO (v/v) to increase solubility. Then, CTS was added to the sample cell, while the tested protein was injected into the syringe. The agitated calorimeter cell, initially holding 5 μM of CTS, was subsequently infused with Keap1 protein at a concentration of 40 μM. The injection procedure was repeated nineteen times based on the built-in programs. Finally, a one-binding site model was used to fit the integrated corrected and concentration-normalized peak regions of the raw data in MicroCal PEAQ-ITC analysis software.

### Histology

Mouse abdominal aortas were collected and embedded in paraffin. 5 µm-thick sections were made by the Fully Automated Rotary Microtome (RM2255, Leica, Wetzlar, Germany). The morphology of the collected aorta was observed using hematoxylin & eosin (H&E) staining following the standard protocol as we previously described 20. Collagen deposition was visualized using Masson's Trichrome. The elastin of aortic sections was also stained with Verhoeff-Van Gieson elastic staining (EVG). Images were acquired with an upright microscope (Olympus, BX53, Tokyo, Japan). Antibodies for immunohistochemical staining were listed in Table S1.

### Western blot analysis

Western blot analysis was performed according to protocols described previously by us 28. Primary antibodies for western blot analysis were listed in Table S1.

### Cell viability assay

Following an overnight seeding of RAVSMCs and MAVSMCs (1 × 10^4^ cells/well) in 96-well plates, a dose gradient of CTS was applied, and the cells were continually incubated for 24 h. As instructed, the MTT kit (C0009S, Beyotime, Shanghai, China) and the CCK-8 kit (CK04, DOJINDO, Tabaru, Japan) were used to assess cell viability.

### Nuclear protein extraction

Following the removal of the culture medium, RAVSMCs were washed three times with cold PBS at the indicated time points. Using the Nuclear and Cytoplasmic Protein Extraction Kit (P0027, Beyotime, Shanghai, China), nuclear and cytoplasmic protein lysates were isolated and collected from RAVSMCs.

### Immunohistochemistry (IHC)

As previously described, paraffin-embedded sections were deparaffinized and rehydrated 29. Sections were heated in Antigen Unmasking Solution (H-3301, Vector Laboratories, CA, USA) at 98 °C for 10 min to retrieve the antigen. Then the slices were blocked and treated with the primary antibodies listed in Table S1 at 4 °C overnight. On the following day, after removing the primary antibody, immunohistochemical staining was carried out using the 3,3'-diaminobenzidine peroxidase substrate kit (SK-4100, Vector Laboratories, CA, USA). All images were captured with an upright microscope (BX53, Olympus Corporation, Tokyo, Japan), and quantitative analysis was conducted using Image J (National Institutes of Health, MD, USA).

### Immunofluorescence

As previously described, frozen blocks were sectioned at 7-µm intervals using a Microm cryostat (CM1950, Leica, Wetzlar, Germany) 28. For cells or frozen sections, cells or slides were washed with PBS, fixed with 4% PFA for 20 min, and permeabilized with 0.3% Triton X-100 solution (0219485483, MP Biomedicals, CA, USA) for 15 min at room temperature. After blocking with 10% normal goat serum (AR0009, Boster, Hubei, China) for 1 h at room temperature, cells or sections were incubated with the primary antibodies listed in Table S1 at 4 °C overnight in a humidified chamber. Samples were then incubated with Alexa Fluor conjugated secondary antibodies (BioLegend, CA, USA) for 60 min at room temperature in the dark after rinsing with PBS. Nuclei were counterstained with DAPI (P0131, Beyotime, Shanghai, China) for 10 min at room temperature in the dark. Images were captured using an inverted laser scanning confocal microscope (LSM800, Carl Zeiss, Oberkochen, Germany).

### RNA isolation and quantitative real-time PCR (qRT-PCR)

Total RNA was collected and extracted from cell cultures and mouse tissue specimens using a TRIzol-based method. qRT-PCR was performed as we previously described 28. The gene expression was quantified using the efficiency-corrected 2^-△△CT^ method using *Gapdh* or *18S rRNA* as the internal control. Primers used in this study were listed in Table S2.

### Zymography (in-situ and gelatin) for matrix metalloproteinases (MMPs) activity detection

**In-situ zymography**: The fluorescein-conjugated gelatin substrate, DQ gelatin (D12054, Invitrogen, CA, USA), was prepared according to manufacturer instructions. Following the addition of the substrate, the 7-µm-thickness frozen sections were incubated for 18 h at 37 °C before taking pictures using a confocal microscope (Axio Observer 3, Zeiss, Oberkochen, Germany). Image J was used to determine the intensity of the fluorescence, and the results were presented as a percentage of the fluorescence area across each cross section.

**Gelatin zymography:** We used gelatin zymography to detect the MMP activity of VSMCs cultured in FBS-free conditioned medium. The conditioned medium of RVASMCs was collected and electrophoreted in a polyacrylamide gel, adding 0.1% (w/v) gelatin. The gels were then washed, stained, and photographed as described previously 17.

### Plasmid transfection

The RAVSMCs were transfected with Keap1^WT^ and Keap1^R415K^ plasmids using Lipofectamine™ LTX reagent (15338100, Invitrogen, CA, USA), following the instructions provided by the manufacturer, respectively. Briefly, 2 μL Lipofectamine™ LTX reagent and 1 μg plasmid were diluted into 50 μL Opti-MEM and incubated at room temperature for 5 min in the presence of PLUS^TM^ reagent. After incubation, the transfection mixture was carefully applied to RAVSMCs in a 24-well cell culture plate, drop by drop. Following a period of 36 h, the cells were subjected to treatment with TNF-α and CTS.

### Seahorse analysis

Aerobic respiration was detected in real time with the Seahorse XFe96 Analyzer (Agilent, CA, USA) by measuring OCR as described previously (Liu, Xu et al. 2020). Briefly, a total of 15,000 cells per well were cultured in XFe96 microplates (103575-100, Agilent, CA, USA). Then pretreat the cells with CTS for 3 h, followed by the addition of TNF-α (10 ng/mL) with an incubation of 24 h. On the following day, the cells were gently rinsed twice with the assay media (Seahorse XF DMEM medium, pH 7.4, 103575-100, Agilent, CA, USA), then switched to the assay medium and prepared for analysis. The Cell Mito Stress Test Kit (103015-100, Agilent, CA, USA) was purchased from Agilent. The final concentration used in the analysis of oligomycin is 1.5 μM, FCCP is 2 μM and rotenone/antimycin is 0.5 μM after optimization. Following measurement, the cells were lysed using RIPA buffer and their O.D. was determined at 562 nm in order to adjust the readings.

### Statistical analysis

All research projects were methodically structured to ensure the formation of groups of equal size through the process of randomization. The researchers carried out a blinded assessment of the trial results and conducted an analysis of the unprocessed data. In order to reduce potential sources of variation, specific outcomes underwent normalization in relation to the control group. GraphPad Prism (Version 8.0.1 for Windows) was used to conduct the statistical analyses. The data were represented as the means ± standard error of the mean (SEM) unless otherwise stated. No outliers were removed from the data set. The variable N represents the number of animals assessed *in vivo* or the number of independent values investigated *in vitro*. A check was conducted to see if the data exhibited normality and equal variance. For comparisons involving more than two groups, either one-way ANOVA or two-way ANOVA followed by Bonferroni's post hoc testing was used. If the data did not pass the aforementioned checks, the Kruskal-Wallis test was used to compare groups consisting of more than two, and the two-stage step-up approach proposed by Benjamini, Krieger, and Yekutieli was applied. The presence of AAA was analyzed using the Fisher exact test. The Kaplan-Meier method was employed to construct survival curves for mice, enabling an analysis of their survival rates. The discrepancies in survival rates were subsequently evaluated using the log-rank (Mantel-Cox) test. Statistical significance was considered as a *P*-value below 0.05.

## Results

### CTS inhibits Ang II-induced AAA formation in ApoE^-/-^ mice

**Figure 1:**
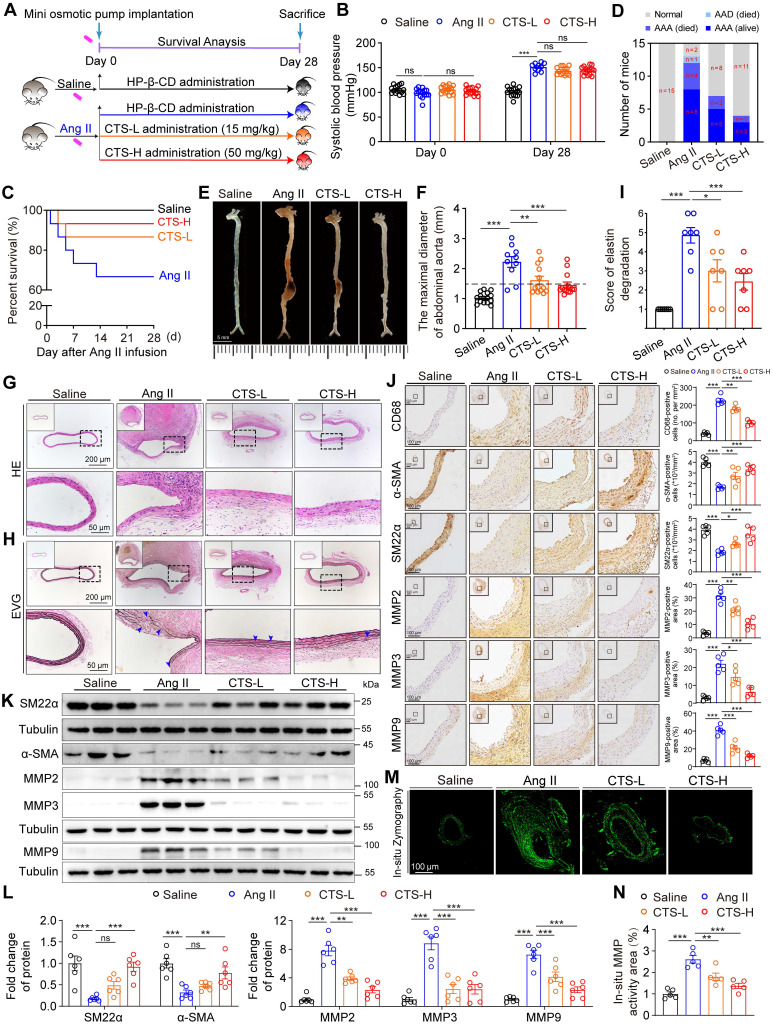
** CTS inhibits Ang II-induced AAA formation in ApoE^-/-^ mice. (A)** Schematic illustration of AAA establishment and CTS administration strategy. In the murine AAA model, 12-week-old male ApoE^-/-^ mice were randomly allocated to four groups and infused with either saline or Ang II (1000 ng/kg/min) for 28 consecutive days. On the day the AAA was started to induce, HP-β-CD (vehicle), low-dose CTS (CTS-L, 15 mg/kg), or high-dose CTS (CTS-H, 50 mg/kg) were intragastrically delivered daily, respectively. (**B**) Systolic blood pressure was measured at the day before Day 0 and Day 28 (n = 10-15). (**C**) Survival rate of AAA. (**D**) Incidence of AAA. (**E**) Representative images of aortas with AAA. (**F**) Maximal diameter of the abdominal aorta was measured in four groups: Sham (n = 15), Ang II (n = 10), CTS-L (n = 13), and CTS-H (n = 14). (**G-H**) Representative images of mouse aorta cross-sections stained with H&E and EVG. In EVG staining, the dark line represents elastin, and blue triangles indicate broken elastin. (**I**) Evaluation of elastin degradation grade. Elastin degradation was graded on a scale of 1-6: grade 1, no degradation; grade 2: elastin degradation less than< 20% of the entire area; grade 3: elastin degradation between 20 and 40%; grade 4: elastin degradation between 40 and 60%; grade 5: elastin degradation between 60 and 80%; and grade 6: elastin degradation ≥80% (n = 7). (**J**) Representative images and quantitative analyses of IHC staining for CD68, α-SMA, SM22α, and MMP2, 3 and 9 (n = 5). (**K-L**) Representative western blots and quantitative analyses of indicated protein expression in mouse aortas (n = 6). (**M-N**) In-situ zymography immunofluorescence images and quantitative analysis for assessing the MMP activities of the cross-sections from mouse aortas. Data are presented as mean ± SEM. *p < 0.05, **p < 0.01, ***p < 0.001, ns: no significant.

To evaluate the effect of CTS on AAA formation, an Ang II-induced murine AAA model was utilized, which is clinically relevant and well-validated 30-32. Twelve-week-old male ApoE^-/-^ mice were administered either low or high doses of CTS (CTS-L or CTS-H) or vehicle daily on the day of Ang II infusion (Figure 1A and Figure S1). CTS was included with HP-β-CD to enhance bioavailability, which has a toroidal structure with a hydrophobic inside and a hydrophilic exterior, forming an inclusion complex 21, 33. At the experimental endpoint, systolic blood pressure was higher in the Ang II-infusion group compared to the saline group, with no notable difference noted with or without CTS treatment (Figure 1B). Five mice experienced premature death in the Ang II group: four from fatal rupture and one from thoracic aortic dissection (AAD). In contrast, only two mice in the CTS-L group and one mouse in the CTS-H group died due to rupture. That is to say, the high dose of CTS remarkably elevated the survival rate in the murine AAA model (Figure 1C-D). Moreover, 80.0% (12/15) of the mice in the Ang II group developed AAA, which was demarcated as a 50.0% increase in the diameter of the aorta. In contrast, only 46.7% (7/15) of the mice in the CTS-L group and 26.7% (4/15) of the mice in the CTS-H group developed AAA (Figure 1D). A significant reduction in the maximal abdominal aortic diameter with the CTS treatment was also observed (Figure 1E-F).

The impairment of the aortic wall integrity aggravates the development of AAA. Therefore, H&E and EVG staining were employed to evaluate the aorta integrity, revealing that CTS substantially alleviated elastin damage and deterioration (Figure 1G-I). Furthermore, the positive staining of CD68 indicated plenty of macrophage infiltration in the Ang-II group, while CTS treatment resulted in a dose-dependent and considerably smaller positive region (Figure 1J). In addition, the downregulation of the contractile markers in VSMCs also contributes to the pathogenesis of AAA 34. Thus, the expression of contractile phenotype markers smooth muscle alpha-actin (α-SMA) and smooth muscle 22α (SM22α) collected from the mouse aortas was evaluated by western blot and IHC. The VSMCs in the middle aortic layer of the Ang II-infused mice exhibited a notable decrease in the levels of α-SMA and SM22α compared with the saline-control mice, while CTS effectively rescued the α-SMA and SM22α decrease (Figure 1J-L). Matrix metalloproteinases (MMPs), including MMP2, 3, and 9, are pivotal in the initiation and progression of AAA through their facilitation of matrix degradation 35, 36. Western blot and IHC both indicated that the levels of indicated MMPs were considerably lower in VSMCs in the aorta media of the CTS-treated mice than those of vehicle-treated control mice (Figure 1J-L). Consistently, in-situ zymography immunofluorescence staining showed substantially lower MMP activities in the aortas of CTS-treated mice compared to Ang II-infused mice (Figure 1M-N). Collectively, these results indicate that CTS hinders the formation of AAA and maintains VSMC homeostasis in the Ang II-induced murine AAA model.

### CTS orchestrates VSMC phenotypes, inflammation, oxidative stress, and mitochondrial function in an *in vitro* AAA model

VSMC dysfunction, including phenotypic switch, inflammation, and oxidative stress, contributes to AAA formation 2. Therefore, we investigated the effects of CTS on the phenotypic switch and the inflammatory and redox status of VSMCs in a TNF-α-induced AAA *in vitro* model. TNF-α was widely used as a stimulant to mimic AAA 20, 37, 38, which has been shown to increase substantially in human and murine AAA 15, 39. 2.5-10 μM CTS showed no toxicity to the rat and mouse VSMCs used in this study Figure S2A-D).

**Figure 2:**
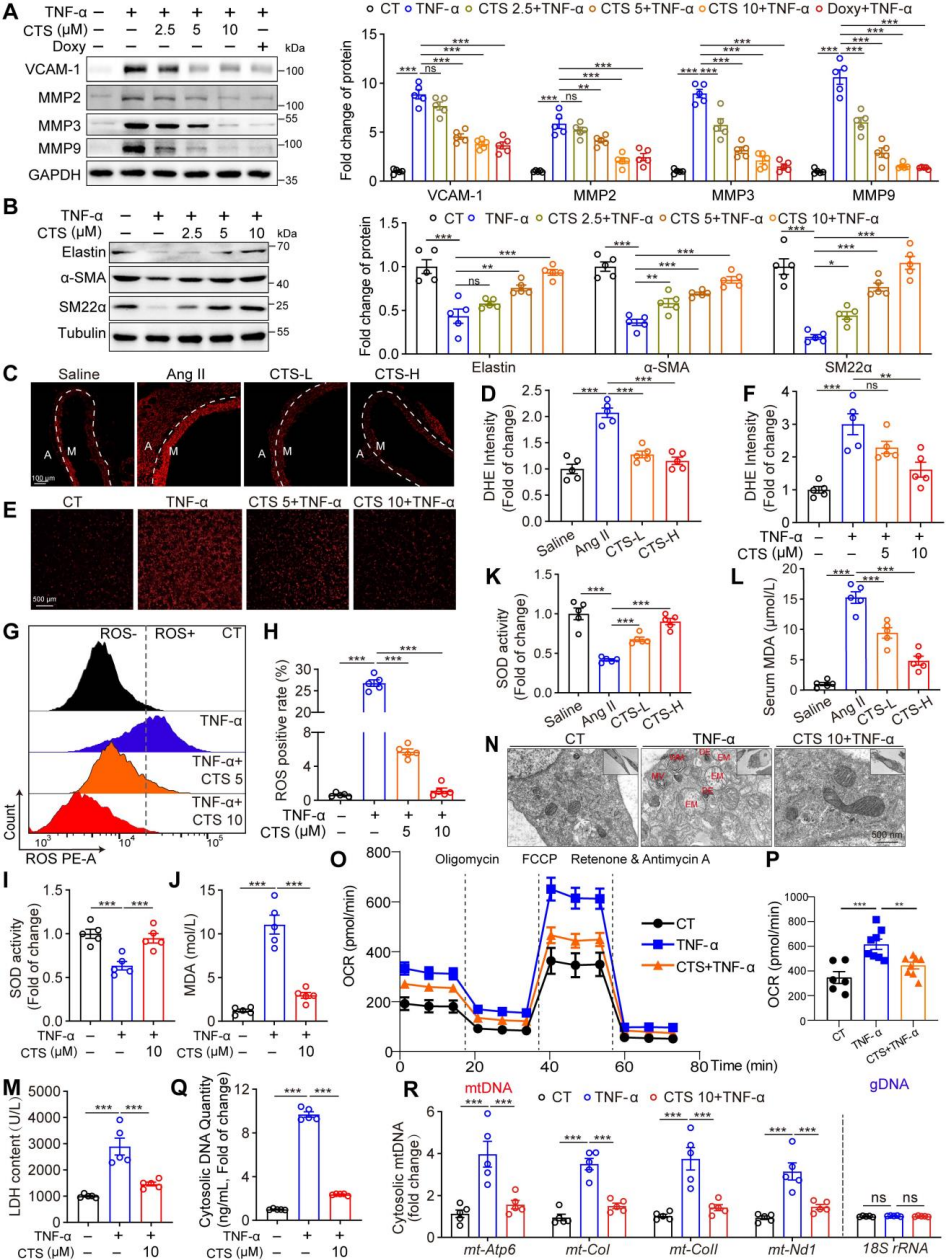
** CTS orchestrates VSMC phenotypes, inflammation, ROS production, and mitochondrial function in an *in vitro* AAA model. (A-B)** Representative western blots and quantitative analyses of the indicated protein expression in RAVSMCs. RAVSMCs were pre-treated with various concentrations of CTS for 3 h, followed by the addition of TNF-α (10 ng/mL) with an incubation of 24 h (n = 5). (**C-D**) Representative images and quantitative analysis of DHE staining in murine abdominal aorta (n = 5) (M: tunica media; A: tunica adventitia) (n = 5). (**E-H**) ROS levels were analyzed through photography (E-F) or flow cytometry (G-H). RAVSMCs were treated as aforementioned in A-B (n = 5). (**I-L**) SOD and MDA levels were measured from mouse serum Ang II-infused mice with or without CTS (low and high doses) treatment (I-J) or from RVASMCs treated as aforementioned in A-B (K-L) (n = 5). (**M**) LDH levels were measured from the cell culture supernatants of RVASMCs (n = 5). (**N**) Representative transmission electron microscope (TEM) images of mitochondria were shown in the RAVSMCs treated with TNF-α and CTS. EM, empty mitochondria; MV, mitochondrial vacuolization; WM, whorl-like inner membrane; DE, dense electron. (**O**) OCR profile of RAVSMCs. (**P**) Quantification of mitochondrial respiration function parameters from O (n = 6-8). (**Q-R**) The quantity of cytosolic DNA and copies of mitochondrial DNA (mtDNA) were measured by qRT-PCR (n = 5) in MAVSMCs. Data are presented as mean ± SEM. *p < 0.05, **p < 0.01, ***p < 0.001, ns: no significant.

Remarkably, CTS dose-dependently suppressed TNF-α-induced protein expression of vascular cell adhesion molecule-1 (VCAM-1) and MMPs (MMP2, 3, and 9), which play critical roles in AAA formation and are mainly responsible for elastin breakdown, in both RAVSMCs (Figure 2A) and MAVSMCs (Figure S2E). Consistently, the proteolytic enzyme activity of MMPs was also inhibited by CTS, as detected by the gelatin zymogram assay (Figure S2F). Doxycycline (DOXY), a broad-spectrum MMP inhibitor that directly binds to the zinc ion domains of MMPs (40, was used as a positive control in the experiments. Furthermore, qRT-PCR demonstrated that CTS downregulated the mRNA expression of MMPs and three classic inflammatory cytokines, *Il1b*, *Il6*, and *Ccl2*, in a dose-dependent manner Figure S2G). As the levels of MMPs, particularly MMP2, are regulated by SMCs that have undergone phenotypic switches (41, we then evaluated the SMC phenotype characteristics in CTS-treated VSMCs. As shown in Figure 2B and Figure S2H-I, CTS increased elastin expression in a dose-related manner while preventing the TNF-α-induced reduction in the expression of SMC contractile phenotypic markers SM22α and α-SMA in both RAVSMCs and MAVSMCs. These observations indicate that CTS hinders the inflammation process in VSMCs, which is a hall marker of AAA, and reduces MMP activities to prevent extracellular matrix degradation and preserve the contractile phenotype of VSMCs.

ROS production is essential in activating MMPs and contributing to AAA formation in both the TNF-α-induced *in vitro* cell model and the Ang II-infusion-induced *in vivo* mouse model 42, 43. As shown in Figure 2C-H, ROS levels, as assessed by DHE staining and flow cytometry, were significantly raised in TNF-α or Ang II treatments, whereas CTS effectively suppressed this raise. Accordingly, the antioxidant superoxide dismutase (SOD) levels and ATP levels were decreased, while the indicator of oxidative stress, malondialdehyde (MDA) levels, were increased in TNF-α-exposed VSMCs and Ang II-treated mice serum, an effect that was prevented by CTS (Figure 2I-L and Figure S2J). In addition, the formation of oxidants by the mitochondrial electron transport chain was examined by detecting the release of mitochondrial H_2_O_2_ using a fluorescent method 24, 44. In the absence of oxidative phosphorylation, respiration in state 4 resulted in a higher release of H_2_O_2_ compared to state 3. This was observed in mitochondria supplemented by both complex I and II substrates. CTS treatment reduced TNF-α-induced H_2_O_2_ production in VSMCs Figure S3A). The inhibitor S1QEL1.1, which hampers the production of superoxide/hydrogen peroxide in the quinone reaction of complex I, substantially reduced this generation. A similar effect was observed by adding rotenone, an inhibitor of the reverse transport generated by succinate (Figure S3B). The release of lactate dehydrogenase (LDH), which is a stable cytoplasmic enzyme, suggested a damaged plasma membrane of the cells (45. In line with the above data, CTS treatment reduced the LDH release in the cell culture supernatant (Figure 2M). The dysfunction of mitochondrial bioenergetics can result in an excessive synthesis of mitochondrial ROS, which are significant contributors to the overall formation of total cellular ROS 46. We speculate that CTS may reduce ROS production via improving mitochondrial dysfunction. To test this hypothesis, we utilized transmission electron microscopy (TEM) to evaluate mitochondrial morphological changes, and used Seahorse extracellular flux analyzer to assess mitochondrial function. As shown in Figure 2N, TEM revealed that, in comparison to the cells in the control group, the mitochondria of the cells following TNF-α treatment exhibited swelling, vacuolation, and nearly total disappearance of the cristae. The matrix of certain mitochondria had electron-dense accumulations and displayed abnormalities in the spiral inner membrane after TNF-α treatment. Following CTS treatment, the cells exhibited a morphology that was close to normal, without obvious ultrastructural damage (Figure 2N). Furthermore, TNF-α treatment induced a significant increase in oxygen consumption rate (OCR) and extracellular acidification rate (ECAR) in VSMCs, while CTS treatment effectively reversed TNF-α-induced mitochondrial dysfunction and glycolysis increase (Figure 2O-P and Figure S3C-D). Overproduction of ROS results in mitochondrial damage and subsequent release of mtDNA, which, in turn, triggers a vicious circle of further ROS and inflammatory factor production 47, 48. Given that CTS was able to suppress ROS production, we postulated that CTS could influence mitochondrial DNA (mtDNA) release. As expected, exposing VSMCs to TNF-α resulted in an augmentation in the quantity of both cytosolic dsDNA and mtDNA, while CTS treatment reversed this effect (Figure 2Q-R and Figure S3E).

Overall, these findings suggest that CTS suppresses VSMC inflammation, ROS production, and mitochondrial damage, thereby maintaining VSMC homeostasis in a TNF-α-induced AAA *in vitro* model.

### Network pharmacology combined with RNA-seq analysis indicates that CTS activates the Keap1-Nrf2-HO-1 pathway and suppresses the cell death pathway

**Figure 3:**
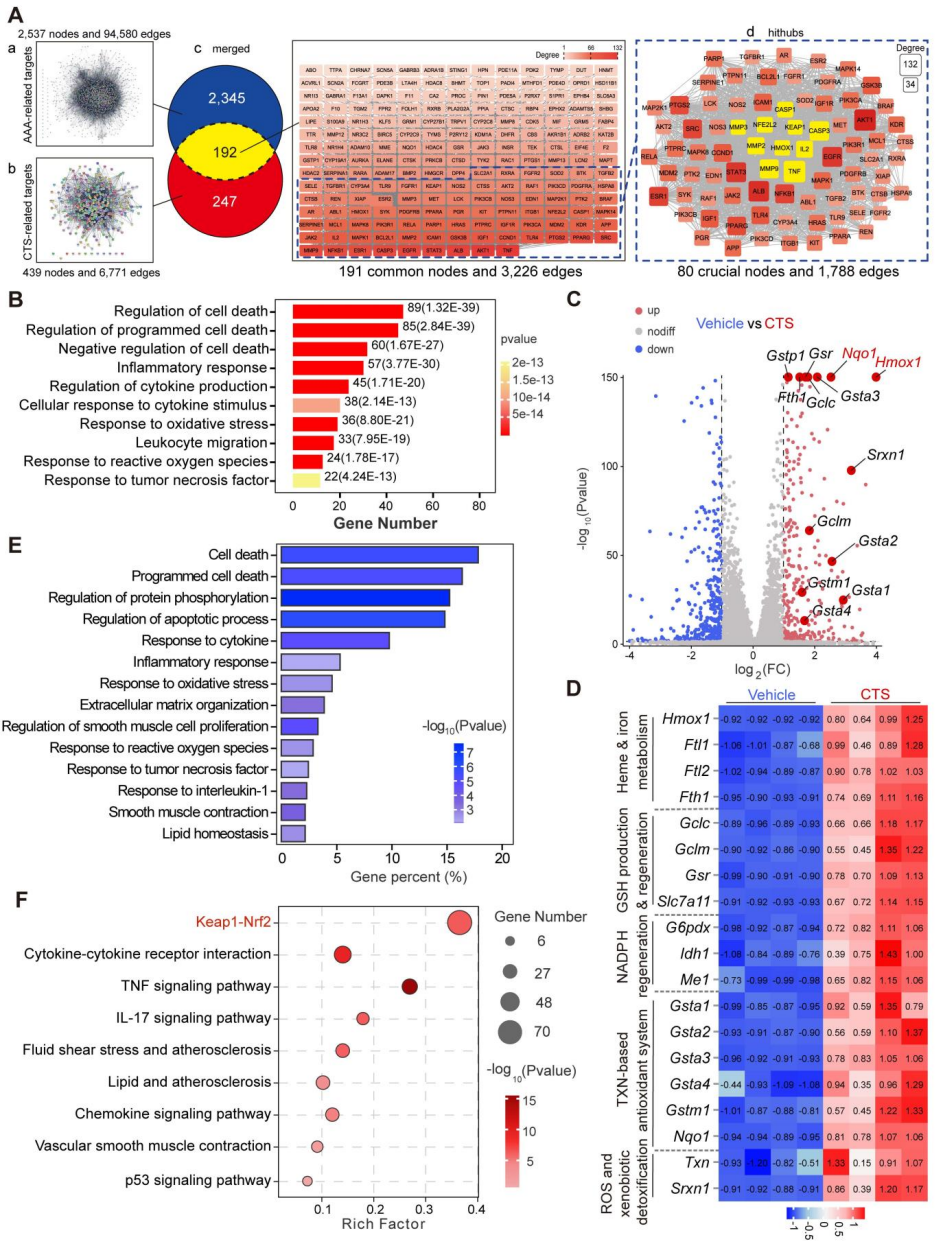
** Network pharmacology combined with RNA-seq analysis indicates that CTS activates the Keap1-Nrf2-HO-1 pathway and suppresses the cell death pathway.** (**A-B**) 2,537 AAA-associated targets and 439 CTS-related targets were collected from free-access databases. Merging these networks revealed 192 common genes, potential targets for CTS in AAA prevention. Cytoscape analysis provided degree values for each target; those exceeding 1.5 times the mean were considered key targets in restraining AAA for CTS. Additionally, the 192 common targets were subjected to DO, KEGG, and GO analyses. (A) The process of screening potential and core targets of CTS in its action on AAA. (B) The DO enrichment analysis revealed that the 192 common genes were primarily associated with inflammatory response and cell death in the biological process ontology. In this analysis, the color and length of the bands were utilized to signify the *P*-value and the percentage of genes. (**C**) For RNA-seq analysis, RAVSMCs were pretreated with 10 μM CTS or DMSO for 3 h, followed by TNF-α treatment for another 3 h. The RNA was then collected and extracted for analysis. Comparing TNF-α treatment to co-treatment with CTS and TNF-α, the volcano plot indicated the magnitude and significance of the changes in gene expression in RAVSMCs. (**D**) The heatmap showed CTS-upregulated genes related to heme and iron metabolism, GSH production and regeneration, NADPH regeneration, the TXN-based antioxidant system, and ROS and xenobiotic detoxification in the RNA-seq analysis. (**E-F**) The KEGG and GO enrichment analyses revealed the effect of CTS on RAVSMCs at the molecular and cellular levels. All DO, KEGG, and GO analyses were performed using the OmicShare tools (https://www.omicshare.com/tools).

To draw a global map of CTS-interacting targets in AAA, network pharmacology was utilized. At first, two distinct target networks were generated: the AAA-target network, which included 2,537 nodes with 94,580 connections, and the CTS-target network, which included 439 nodes with 6,771 connections (Figure 3A a-b). The overlapping network was formed by combining the two networks with CTS and AAA as cores, respectively, which comprised 191 nodes (The TTPA node was hidden due to no connection to any other targets) and 3,226 connections. To identify the most potential key nodes in the overlapping network, only the nodes whose degree values were 1.5 times or higher than the median value of 191 common nodes were focused, resulting in a new hithub network with 80 crucial nodes. Within this focused network, ten targets marked in yellow (KEAP1, NFE2L2, HMOX1 49; MMP2,3,9 50; CASPASE1 51, IL2 52, TNF 15, and CASPASE3 53) are vital in AAA and have been shown to be closely linked to the progress of AAA, particularly the Keap1-Nrf2-HO-1 signaling axis (Figure 3A c-d).

To extensively explore the underlying mechanisms of CTS in preventing AAA, enrichment analyses including DO, KEGG, and GO on the 192 common targets were performed. At first, the DO analysis surprisingly showed that 19 out of the top 20 items were linked to human vascular disorders, strongly suggesting the potential of CTS in the management of vascular diseases, including AAA Figure S4A). Furthermore, based on the KEGG functional enrichment analysis, the 192 intersection targets were remarkably enriched in pathways including “Lipid and atherosclerosis”, “Fluid shear stress and atherosclerosis” and “TNF signaling pathway”, which have been proven to be closely linked to AAA (Figure S4B) (54, 55. Moreover, according to the GO analysis results, CTS was shown to have functions in multiple cellular regions and affect multiple vital biological activities via interacting with other proteins Figure S4C). Notably, the predominantly enriched pathways of CTS were focused on the categories of inflammatory response and cell death (Figure 3B).

Considering that inflammation and cell death are crucial characteristics of AAA (9, we hypothesized that CTS could potentially inhibit AAA development through its anti-inflammatory and cell death repression effects. To further specify the role of CTS in AAA in a nonbiased manner, RNA-seq was performed on RAVSMCs, which is a widely used primary culture in vascular research, including in studies focusing on aneurysm 56, 57. The volcano plot showed an increase in target genes regulated by the transcription factor Nrf2 when exposed to CTS, with *Hmox-1* (gene name of HO-1) exhibiting the most notable upregulation compared to other genes (Figure 3C). Among the Nrf2 target proteins, HO-1 is a key cytoprotective enzyme that possesses potent anti-inflammatory and antioxidant properties 58, as well as MMP inhibitory effects 59. The protein levels of HO-1 are shown to be associated with the severity of AAA in patient specimens 60, and *Hmox-1* knockout mice are more vulnerable to developing Ang II-induced AAA 61. To further investigate the possible significance of the Nrf2-HO-1 pathway as a crucial target for CTS in preventing AAA, we treated critical AAA-associated cells, including VSMCs, macrophages (MΦ), and ECs, with CTS. Western blot analysis showed that CTS upregulated HO-1 expression in VSMCs, MΦ, and ECs in a dose-dependent manner, with the most pronounced effect observed in VSMCs Figure S4D). Furthermore, a heatmap was generated to clearly show the high transcription of Nrf2 target genes in the CTS treatment group compared to the vehicle group (Figure 3D). In addition, the KEGG analysis of differentially expressed genes showed that CTS has a notable impact on the regulation of inflammation and cell death pathways, consistent with the findings of network pharmacology (Figure 3E). In the GO enrichment analysis, it became evident that CTS exerted its influence on the “Keap1-Nrf2 pathway,” aligning with the results of network pharmacology (Figure 3F).

### CTS activates the Keap1-Nrf2-HO-1 pathway and suppresses pyroptosis pathway in VSMCs to prevent AAA

**Figure 4:**
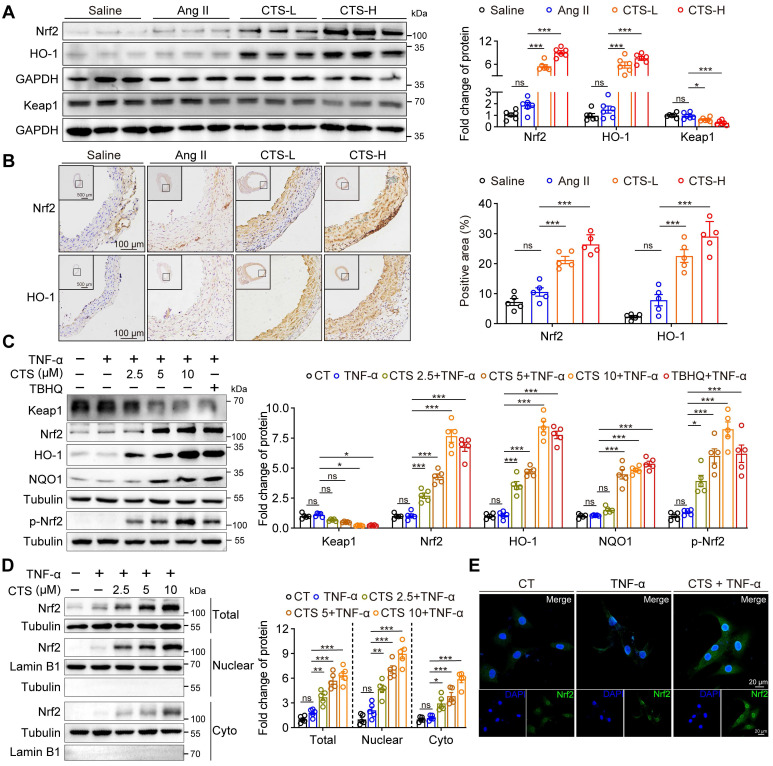
** CTS activates the Keap1-Nrf2-HO-1 pathway in VSMCs to prevent AAA.** (**A**) Representative western blots and quantitative analysis of indicated protein expression in mouse aortic tissues from Ang II-infused mice with or without CTS (low and high doses) treatment (n = 6). (**B**) Representative images and quantitative analyses of IHC staining for Nrf2 and HO-1 from the cross-sections of the mouse abdominal aorta (n = 5). (**C**) Representative western blots and quantitative analyses of the indicated protein expressions in RAVSMCs treated with different doses of CTS (2.5, 5 and 10 μM). TBHQ is an activator of the Nrf2 pathway used as a positive control (n = 5). (**D**) Representative western blot analysis and quantitative assessment of Nrf2 in RAVSMCs (n = 5). (**E**) Immunofluorescence staining showed the distribution and colocalization of Nrf2 (green) in RAVSMCs treated with TNF-α and CTS or DMSO. Data are presented as mean ± SEM. *p < 0.05, **p < 0.01, ***p < 0.001, ns: no significant.

To further explore the effect and regulation mechanism of CTS in alleviating VSMC inflammation and oxidative stress during the development of AAA, we draw from the data shown in Figure 3 that the Keap1-Nrf2-HO-1 pathway and cell death regulation are crucial in preventing AAA with CTS. In line with the findings of network pharmacology and RNA-seq, CTS treatment induced a robust protein expression of Nrf2 and HO-1 in VSMCs in the aorta media of Ang II-infused mice *in vivo* (Figure 4A-B). Further analysis of the CTS effect on cultured VSMCs *in vitro* revealed a decrease in Keap1 protein expression and a marked increase in Nrf2 and its target proteins, HO-1 and NQO1 (Figure 4C and Figure S5A), and the effects of CTS (10 μM) were comparable with tert-butylhydroquinone (TBHQ), one of the most potent Nrf2 activity inducers (62. Keap1 degradation and Nrf2 translocation to the nucleus are essential steps for activating the Nrf2-HO-1 pathway. Furthermore, qRT-PCR demonstrated that CTS upregulated the mRNA expression of Nrf2 target genes, *Hmox-1*, *Gclc*, *Gclm*, *Nqo1*, *Fth1*, and *Ftl1* in a dose-dependent manner Figure S5B). Furthermore, our research showed that CTS significantly raised the expression of Nrf2 and HO-1 protein in RAVSMCs and MAVSMCs compared to other tanshinone monomers (Figure S5C). We observed an up-regulation in the protein expression of total Nrf2. Subsequent investigations indicated that the protein levels of Nrf2 were increased in both the nucleus and cytoplasm, with the increase being more pronounced in the nucleus (Figure 4D). This was further confirmed by immunofluorescence staining (Figure 4E), indicating a notable activation of the Nrf2 pathway.

**Figure 5:**
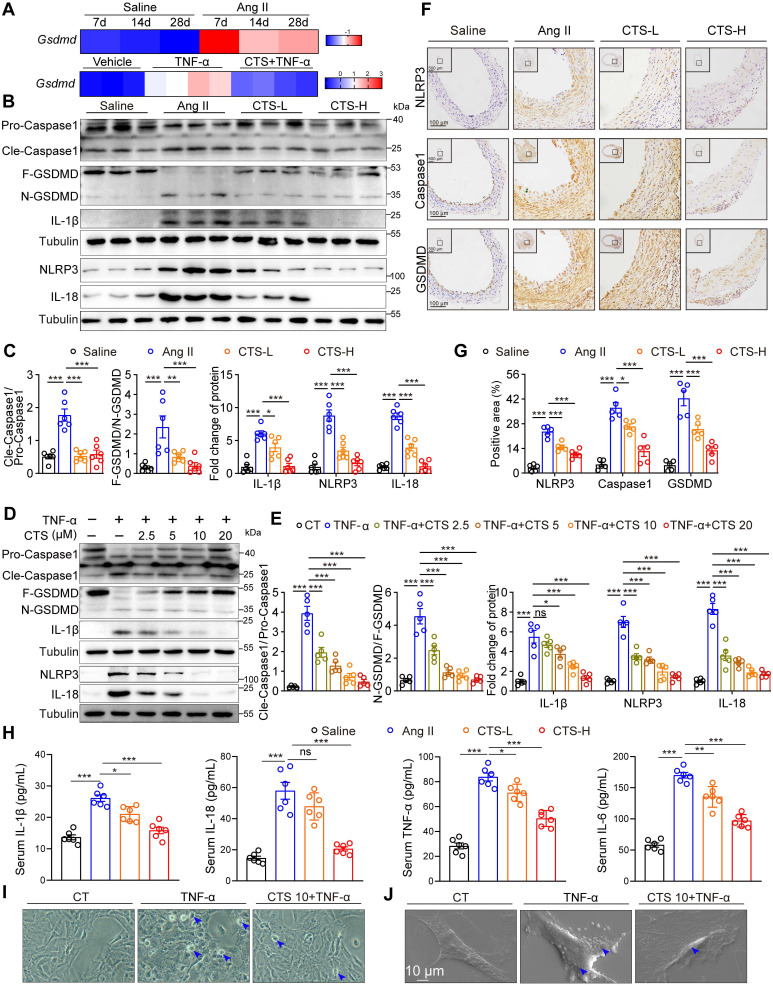
** CTS suppresses VSMC pyroptosis to prevent AAA.** (**A**) Heatmap showing the gene expression of *Gsdmd* in the aortas of Ang II/Saline-infused mice from 7 to 28 days. The data are from a reanalysis of the reported microarray dataset (GSE17901). n = 5-7 (upper); Heatmap showing the gene expression of *Gsdmd* in RAVSMCs treated with TNF-α and CTS (n = 3-4) (lower). (**B-E**) Representative western blots and quantitative analysis of indicated protein expression in mouse aortic tissues from Ang II-infused mice with or without CTS treatment, as well as RAVSMCs treated with TNF-α and CTS (n = 5-6). (**F-G**) Representative images and quantitative analyses of IHC staining for NLRP3, Caspase 1, and GSDMD (n = 5). (**H**) Cytokines in mouse serum from the indicated groups were measured by ELISA (n = 6). (**I**) Representative phase-contrast imaging assays were performed. Arrowheads indicated balloon-like pyroptotic cells in the RAVSMCs treated with TNF-α and CTS. (**J**) Representative scanning electronic microscopy (SEM) images of pyroptosis were shown in the RAVSMCs treated with TNF-α and CTS. Data are presented as mean ± SEM. *p < 0.05, **p < 0.01, ***p < 0.001, ns: no significant.

In addition to the Keap1-Nrf2 pathway, network pharmacology and RNA-seq analysis also revealed a considerable enrichment of cell death-related pathways simultaneously. Accumulating evidence indicates that VSMC pyroptosis, a form of programmed cell death that is triggered by inflammation, contributes to the development of AAA (10-12. NLRP3, a crucial sensor of inflammasomes, plays a key role in the recruitment, cleavage, and activation of pro-Caspase 1, leading to the formation of the inflammasome and subsequent initiation of pyroptosis. Activated Caspase 1 results in the cleavage of GSDMD, a key executive protein in pyroptosis, the insertion of its N-terminal segment into the plasma membrane to form pores, and the subsequent release of IL-1β and IL-18 63. Elevated mRNA levels of *Gsdmd* were observed in the aorta tissues of the Ang II-infused mice from 7 to 28 days compared to saline controls (Figure 5A). Also, our RNA-seq data showed the transcription level of *Gsdmd* was elevated in the TNF-α-stimulated VSMCs, while CTS treatment alleviated this elevation (Figure 5A). The protein levels of the key targets in the pyroptosis pathway, including NLRP3, Cle-Caspase1, N-GSDMD, IL-1β, and IL-18, were detected by western blot and showed a significant increase either in the Ang II-infused mouse aorta tissues or TNF-α-treated RAVSMCs (Figure 5B-E) and MAVSMCs Figure S6 while inhibited by CTS. In addition, the expression of NLRP3, Caspase1, and GSDMD in the cross-sections of the mice aortas was confirmed by IHC (Figure 5F-G), and the release of IL-1β, IL-18, and other two critical AAA-related cytokines (TNF-α and IL-6) in the mice serum was confirmed by ELISA assays (Figure 5H). Lastly, morphological changes of pyroptosis were observed by phase-contrast microscope and SEM. Under the phase-contrast microscope, the cells exhibited balloon-like morphology. Furthermore, clear indications of the swelling of cell size and pores forming on the cell membrane were observed by SEM in VSMCs when stimulated with TNF-α, while the treatment of CTS improved the abnormal morphology (Figure 5I-J).

### CTS directly binds to the Arg415 residue of Keap1 to activate the Nrf2 pathway and improve oxidative stress and inflammation

**Figure 6:**
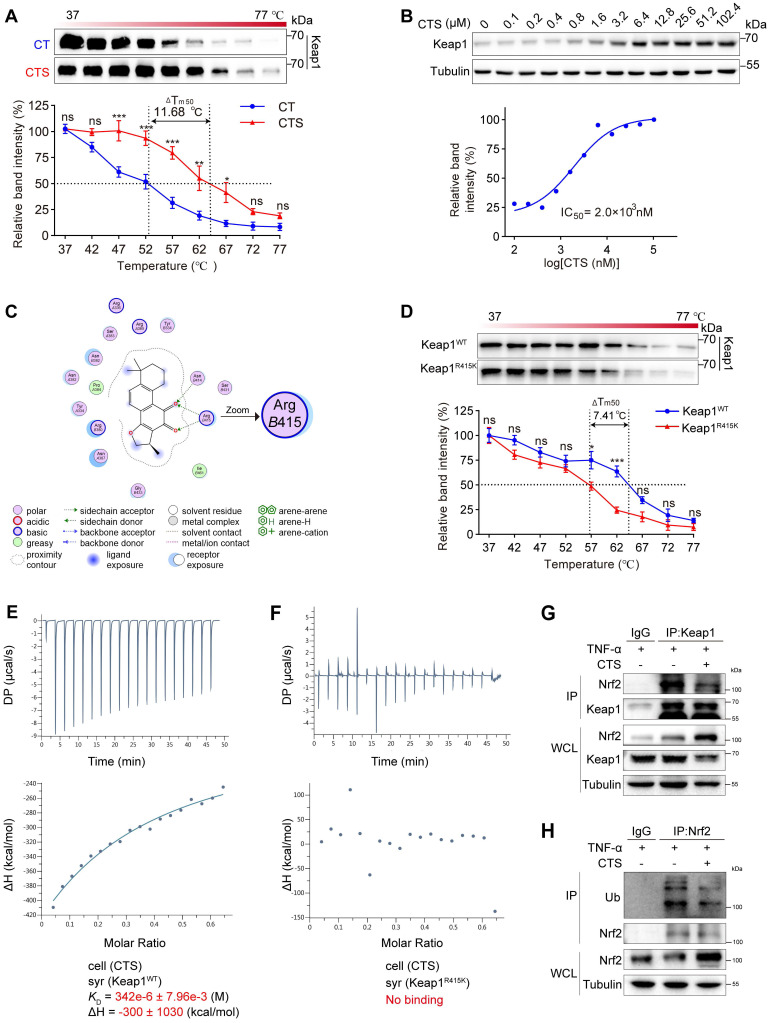
** CTS directly binds to the Arg415 residue of Keap1 to activate Nrf2.** (**A**) The CETSA was performed using intact RAVSMCs with 10 μM of CTS. The stability of Keap1 protein at 37-77 °C was measured by western blot. (**B**) The ITDRF-CETSA assay was performed using intact RAVSMCs in the presence of different CTS doses (0-102.4 μM) over 4 h. The stability of Keap1 protein at 55 °C was measured by western blot. (**C**) The 2D image illustrates the interaction of CTS and Keap1 at specific amino acids. Hydrogen bonds are denoted by green and blue dotted lines. (**D**) The CETSA was performed using purified Keap1 WT/R415K proteins in the presence of CTS (10 μM). The stability of Keap1 proteins under 37-77 °C was measured by western blot. (**E-F**) Isothermal titration plot of 2 μM CTS (in sample cell) with 40 μM Keap1 WT or Keap1 R415K proteins (in syringe). The inset provided a graphical representation of the thermodynamic parameters, including KD and ΔH. The solid line represents the optimal nonlinear least-squares fit to a single binding site model. (**G**) Co-immunoprecipitation (Co-IP) of Keap1 in RAVSMCs treated with TNF-α (10 ng/mL) and CTS (10 μM) or DMSO to detect the protein level of Nrf2. (**H**) Co-IP of Nrf2 in RAVSMCs treated with TNF-α (10 ng/mL) and CTS (10 μM) or DMSO was performed to detect the ubiquitination level of Nrf2. WCL: whole cell lysates. Data are presented as mean ± SEM. *p < 0.05, **p < 0.01, ***p < 0.001, and ns: no significance.

Keap1 exerts strict control over the activity of Nrf2. Under stress or in the presence of Nrf2 activators, Nrf2 disconnects from Keap1, avoids ubiquitination and subsequent degradation, translocates into the nucleus, and transcripts a collection of genes to prevent oxidation and inflammation 64. To gain more structural insight into how CTS affects the Keap1-Nrf2 pathway, we used CETSA, molecular docking analysis, and ITC to determine whether there is a direct contact between CTS and Keap1. Firstly, the binding between Keap1 protein and CTS was evaluated by the CETSA assay performed with VSMCs, which demonstrated that CTS had a substantial effect on increasing the thermal stability of Keap1 with a Tm50 of 11.68 °C (Figure 6A). In addition, the ITDRF-CETSA assay provided further proof of the dose-dependent binding and significant stabilization of the Keap1 protein by CTS with an IC50 of 2 μM (Figure 6B). Secondly, we utilized the MOE software platform 65 to identify the direct binding sites between Keap1 and CTS, and the molecular docking analysis displayed that CTS had the highest affinity for the Arg415 (R415) residue of Keap1 (Figure 6C). The Arg415 residue is situated in the side chain of Keap1 and remains the most significant contribution to binding energy at the Keap1-Nrf2 interface via forming a hydrogen bond 66.

To confirm that Arg415 is the competitive binding site of Keap1 with CTS and Nrf2, the amino acid arginine was substituted with lysine (Lys415, K415) other than alanine or glycine to maintain a similar charged environment 67. The binding capacity between Keap1 R415K mutated protein and CTS was evaluated by CETSA and showed a notable decline in thermal stability of Keap1 R415K protein with CTS, with a Tm50 value of 7.41 °C compared to Keap1 WT protein (Figure 6D). Subsequently, ITC assays were also performed to further examine the binding affinity between these two Keap1 proteins and CTS and revealed that CTS exhibited a higher binding affinity for the Keap1 WT protein, while no evident binding was observed for the mutated Keap1 (Figure 6E-F). Furthermore, Co-immunoprecipitation (Co-IP) assays revealed that CTS treatment significantly reduced the Keap1 protein levels combined with Nrf2 in VSMCs (Figure 6G). We also observed that the ubiquitination level of Nrf2 was reduced after CTS treatment (Figure 6H). These findings suggest that CTS directly interact with the Arg415 residue in Keap1, thereby obstructing the binding between Keap1 and Nrf2, blocking Nrf2 ubiquitination and subsequent degradation, resulting in the Nrf2 escape and translocation to the nucleus to start transcription of cytoprotective genes.

**Figure 7:**
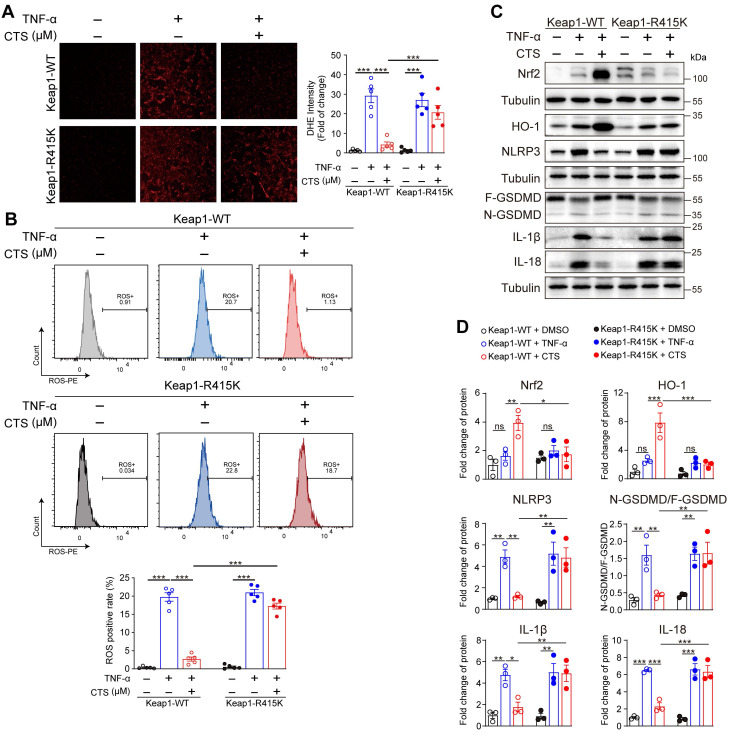
CTS binds to Keap1 at Arg415 to reduce the generation of ROS via activating Nrf2 and inhibiting pyroptosis in TNF-α treated VSMCs. (**A**-**B**) ROS levels were analyzed through photography (A) or flow cytometry (B). RAVSMCs were transfected with Keap1-WT and Keap1-R415K plasmids, respectively. After 36 h, the transfected cells were pretreated with CTS for 3 h followed by the addition of TNF-α (10 ng/mL) with an incubation of 24 h. Cells were stained and collected for photography or flow cytometry analysis. (**C-D**) Representative western blot analysis and quantitative assessment of indicated proteins in RAVSMCs (n = 3). Data are presented as mean ± SEM. *p < 0.05, **p < 0.01, ***p < 0.001.

To confirm our hypothesis that CTS facilitated the dissociation of Nrf2 and Keap1 by competitively binding with Keap1 at Arg415, VSMCs were transfected with Keap1 R415K mutated plasmid (Keap1-R415K) or Keap1 WT vector (Keap1-WT). DHE staining and flow cytometry revealed that ROS levels were significantly improved after CTS treatment in VSMCs transfected with Keap1-WT, but not in Keap-R415K transfected cells (Figure 7A-B). Additionally, transfection of Keap1^R415K^ into VSMCs significantly inhibited CTS-induced upregulation of Nrf2 and HO-1 protein expression (Figure 7C-D). Moreover, western blot showed that CTS possessed anti-inflammatory and anti-pyroptosis effect in VSMCs transfected with Keap1^WT^, but not Keap1^R415K^ (Figure 7C-D). In summary, the binding of CTS to the amino acid at site-415 in Keap1 is critical for ameliorating oxidative stress, promoting the activation of Nrf2-mediated signaling pathways, and inhibiting inflammatory response in VSMCs.

### Inhibition of the Keap1-Nrf2 pathway reverses the protective effect of CTS in an *in vitro* AAA model

**Figure 8:**
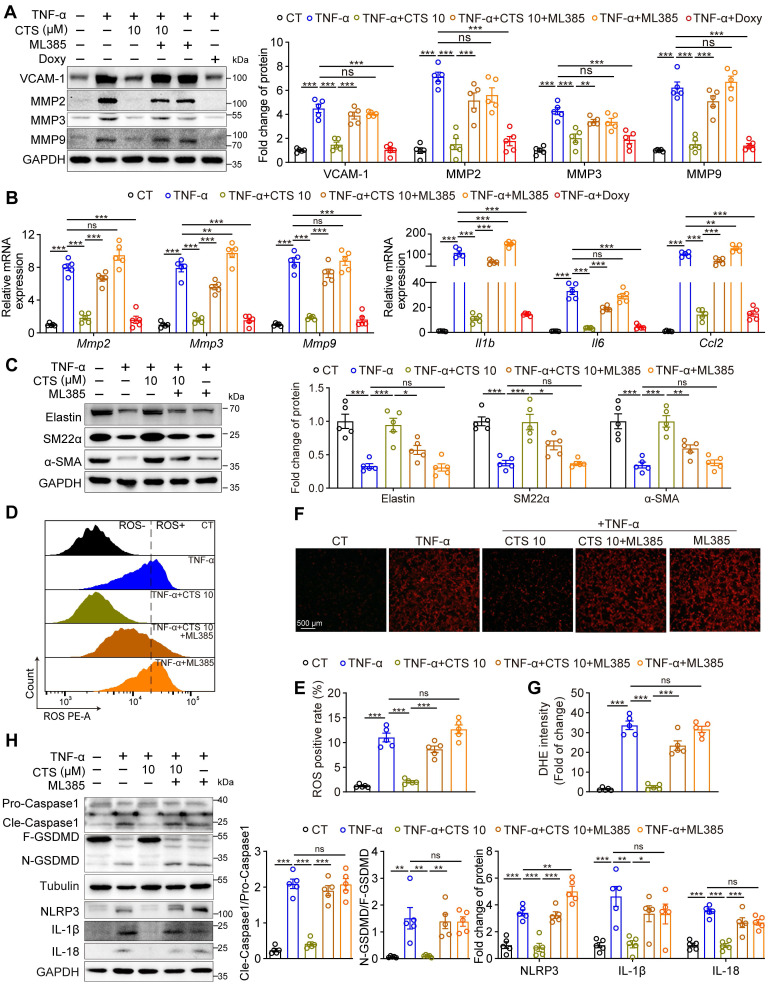
** Nrf2 inhibition reverses the protective effect of CTS *in vitro*. (A)** Representative western blots and quantitative analysis of indicated protein expression in RAVSMCs treated with TNF-α, CTS, and ML385. ML385 was used as an inhibitor of Nrf2 (n = 5). **(B)** qRT-PCR analysis of the mRNA expression of MMPs and cytokines in RAVSMCs treated with TNF-α, CTS, and ML385 (n = 5).** (C)** Representative western blots and quantitative analysis of indicated protein expression in RAVSMCs treated with TNF-α, CTS, and ML385 (n = 5). **(D-G)** ROS levels were analyzed through flow cytometry (D-E) or photography (F-G) in RAVSMCs treated with TNF-α, CTS, and ML385 (n = 5). **(H)** Representative western blots and quantitative analysis of indicated protein expression in RAVSMCs treated with TNF-α, CTS, and ML385 (n = 5). Data are presented as mean ± SEM. *p < 0.05, **p < 0.01, ***p < 0.001, and ns: no significance.

Our observations that CTS significantly activates the Keap1-Nrf2 pathway and attenuates AAA formation prompted us to explore whether Nrf2 activation underlies CTS-mediated protective effects on AAA by using the Nrf2 transcriptional inhibitor ML385 68. As shown in Figure 8A, the elimination of MMPs (MMP2, 3, and 9) along with VCAM-1 protein levels by CTS was completely abolished in the presence of ML385. A similar result in the mRNA levels of MMPs and AAA-related cytokines (*Il1b*, *Il6*, and *Ccl2*) was observed (Figure 8B). In addition, the ability of CTS to restore the protein levels of elastin and SMC contractile phenotypic markers (SM22α and α-SMA) was partially compromised by ML385 (Figure 8C). CTS-mediated antioxidant capabilities were also reversed by ML385 treatment, as indicated by the detection of ROS levels using flow cytometry (Figure 8D-E) and DHE staining (Figure 8F-G). Furthermore, the inhibitory effect of CTS exerted on NLRP3-Cle-Caspase 1-N-GSDMD-mediated pyroptosis was abrogated by ML385 administration (Figure 8H). Taken together, these results indicate that CTS maintains VSMC homeostasis and suppresses VSMC pyroptosis in AAA in an *in vitro* model by activating Nrf2.

### VSMC-specific *Nfe2l2* knockdown abolishes the protective effect of CTS in an *in vivo* murine AAA model

**Figure 9:**
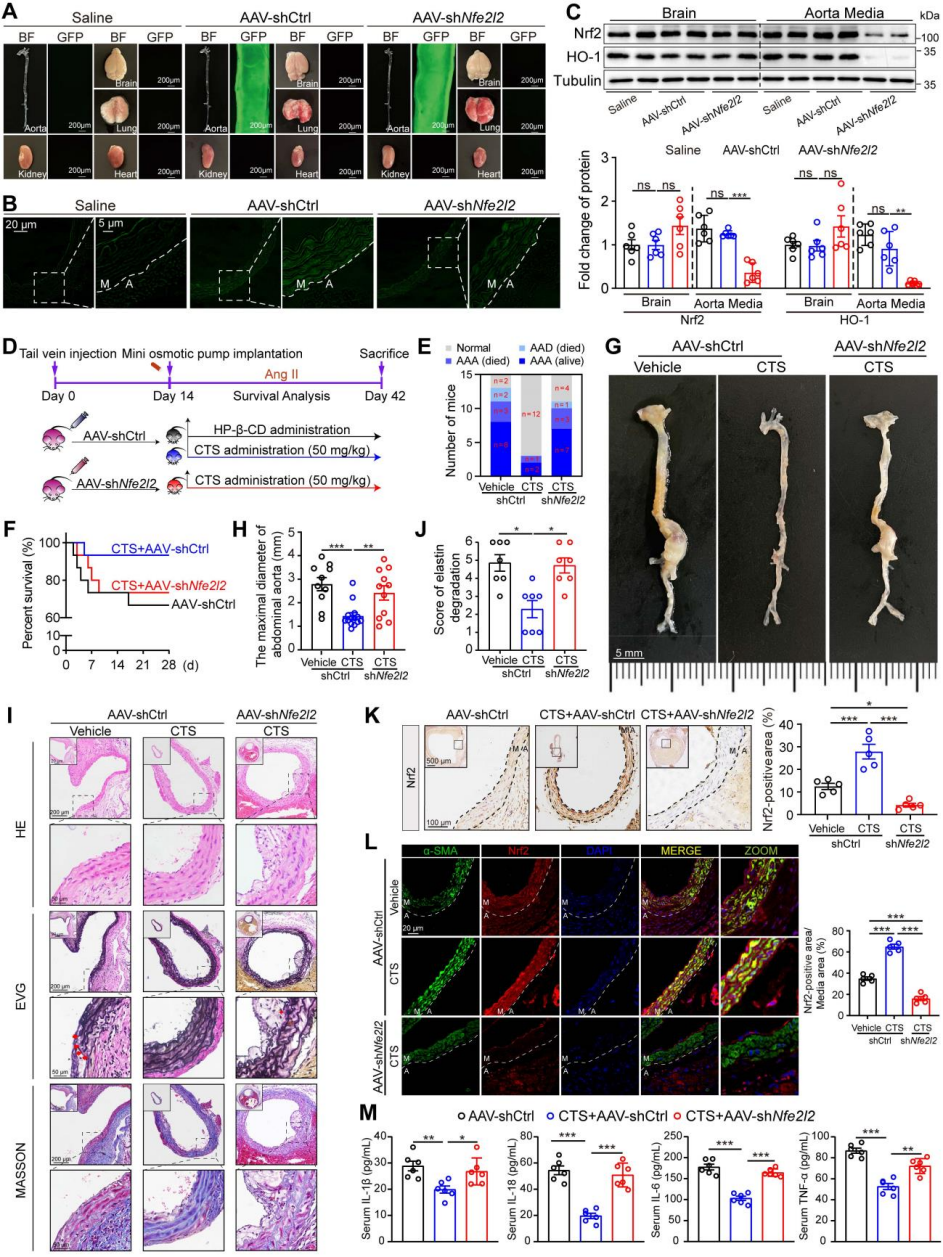
** VSMC-specific *Nfe2l2* knockdown abolishes the protective effect of CTS in Ang II-induced mouse AAA model.** (**A**) Mouse organs were collected for GFP-fluorescence detection. Both bright-field and fluorescence images were captured under the same conditions. (**B**) Representative images of fluorescence imaging of mouse aorta across sections (M, media; A, adventitia). (**C**) Representative western blots and quantitative analysis of indicated protein expression in mouse brains and aortas. n = 5. (**D**) Schematic illustration of SMC-specific *Nfe2l2* knockout in mice, AAA model establishment, and CTS administration strategy. Ten-week-old male ApoE^-/-^ mice were tail-vein-injected with AAV2-shCtrl and AAV2-sh*Nfe2l2* viruses, respectively. After two weeks, all the mice were infused with Ang II (1000 ng/kg/min) to establish the AAA model. On the day the AAA was started to induce, HP-β-CD (vehicle) or high-dose CTS (50 mg/kg) were intragastrically delivered daily, respectively. At the endpoint, blood, aorta, and other organs were collected for the following experiments. (**E**) Percent survival. (**F**) AAA incidence. (**G**) Representative images of abdominal aortic aneurysm. (**H**) Maximal diameter of the abdominal aorta. (**I**) Representative images and quantitative analysis (for elastin) of vessel cross-sections stained with H&E, EVG, and Masson's trichrome staining. (**J**) Quantitative analysis of elastin degradation related to EVG staining in panel I. (**K**) Representative images and quantitative analysis of immunohistochemical staining for Nrf2. (**L**) Images and quantitative analysis of Nrf2 and α-SMA immunofluorescence in the murine abdominal aorta. (**M**) Cytokines from mouse serum were measured by ELISA kits (n = 6). Data are presented as mean ± SEM. *p < 0.05, **p < 0.01, ***p < 0.001, and ns: no significance.

**Figure 10:**
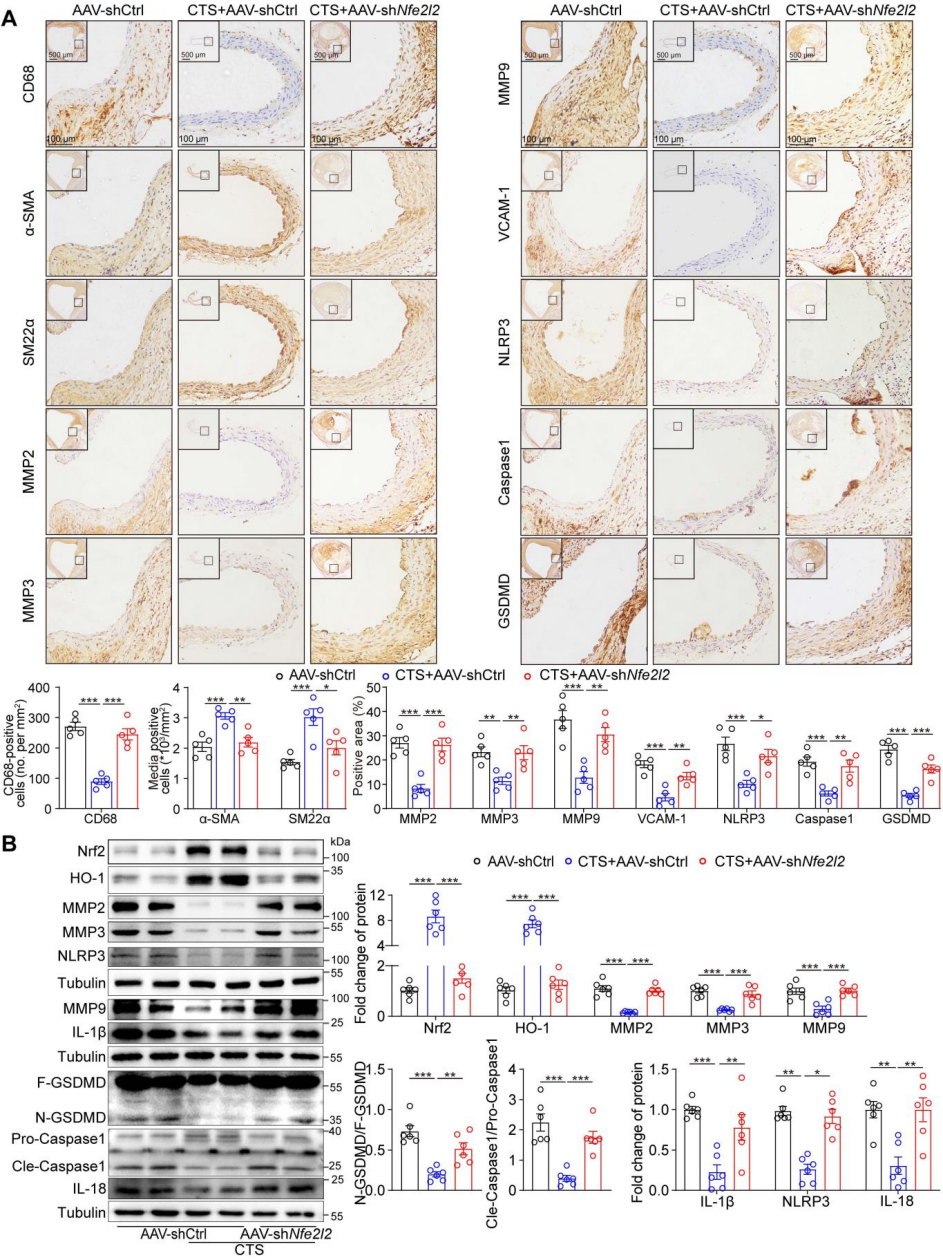
** SMC-specific *Nfe2l2* knockdown abolishes the effect of CTS on anti-inflammatory, antioxidant, and inhibiting MMP activity and generation *in vivo*.** (**A**) Representative immunohistochemical staining and quantitative analysis of indicated proteins in mouse aorta cross-sections (n = 5). (**B**) Representative western blots and quantitative analysis of indicated proteins in murine aorta (n = 6). Data are presented as mean ± SEM. *p < 0.05, **p < 0.01, ***p < 0.001, and ns: no significance.

To further confirm the role of the Keap1-Nrf2 pathway in VSMCs as a vital target for CTS *in vivo*, the *Nfe2l2* shRNA (sh*Nfe2l2*) sequence conjugated with GFP was cloned into the AAV2 vector driven by SM22α, particularly targeting VSMCs. We first validated the effect of AAV-mediated gene knockdown in the ApoE^-/-^ mice. Mice were injected with the control virus (AAV-shCtrl) and AAV-sh*Nfe2l2* via the tail vein. After two weeks, fluorescence imaging revealed that the AAV specifically infected the aorta rather than other organs (Figure 9A). Cross-sections of the mouse aorta showed the GFP was specifically expressed in the media tunica (Figure 9B). Moreover, the western blot analysis indicated that the Nrf2 protein expression in the brain was consistent in the presence of AAV2 viruses, while there was a notable decrease in the aorta (Figure 9C). After validating the effectiveness of AAV-sh*Nfe2l2*, we proceeded to inject the viruses into ApoE^-/-^ mice to establish the Ang II-induced mouse AAA model aforementioned (Figure 9D). As expected, we observed that CTS effectively suppressed AAA formation in Ang II-infused ApoE^-/-^ mice. Intriguingly, the inhibitory impact was diminished when Nrf2 was silenced in mouse VSMCs, as indicated by a rise in the incidence of AAA, a decrease in survival, and an increase in the maximum diameter of the abdominal aorta (Figure 9E-H). Additionally, histological evaluations depicted a direct connection between Nrf2 deficiency and compromised CTS therapeutic efficacy. This was manifested by notable adventitial thickening, increased elastin fragmentation, and a considerable reduction in collagen content within the medial layer of the aorta (Figure 9I-J). Immunohistochemical analyses corroborated these findings, demonstrating that AAV-mediated sh*Nfe2l2* effectively inhibited CTS-induced Nrf2 stabilization and activation in VSMCs (Figure 9K). Further immunofluorescence studies revealed that CTS administration substantially upregulated Nrf2 expression in VSMCs. This upregulation was impeded by employing AAV-sh*Nfe2l2*, which concurrently led to a reduction in α-SMA protein levels (Figure 9L). Cytokines, including IL-1β, IL-6, IL-18, and TNF-α, were detected by ELISA and revealed a distinguished decrease in the CTS-treated mice compared to Ang II-infused mice. However, the treatment effects of CTS were largely abrogated by Nrf2 knockdown (Figure 9M). Furthermore, immunohistochemical staining showed that Nrf2 knockdown in VSMCs blocked the therapeutic effect of CTS by increasing inflammation, promoting VSMC phenotypic switch, and raising MMP levels. This was observed even though CTS treatment could improve AAA by activating Nrf2-mediated pathways and elastin levels (Figure 10A), highlighting Nrf2 as a key therapeutic target in AAA treatment. Western blot analysis further proved that the activation of the Nrf2 pathway and suppression of the NLRP3 and pyroptosis pathways were greatly weakened by the Nrf2 knockdown (Figure 10B). Taken together, these data suggest that CTS is promising in treating AAA by activating Nrf2-mediated anti-inflammatory effects and suppressing NLRP3-caspase1-initiated pyroptosis.

## Discussion

In this study, CTS has been identified as a potential pharmacological therapy for AAA, a condition that currently lacks medication-based therapeutic choices in clinical practice. CTS effectively decreased the incidence of AAA, reduced the maximum diameter of the abdominal aorta, and increased the survival rate in Ang II-infused ApoE^-/-^ mice, a well-established and clinically relevant model for studying AAA. Mechanistically, we demonstrated that CTS inhibited VSMC inflammation and preserved the VSMC contractile phenotype by suppressing VSMC pyroptosis and ROS production via activation of the Keap1-Nrf2 pathway. Molecular docking, CETSA, and ITC combined with site-directed mutagenesis revealed that CTS selectively binds to the Arg415 (R415) residue of Keap1 and facilitates the separation of Nrf2 from Keap1.

No pharmaceutical therapy for AAA has sparked substantial interest in understanding the pathogenesis of this condition. Most prior research in animals and humans revealed the vital role of chronic inflammation in AAA pathogenesis 2. For example, two very recent studies have reported that the widely recognized medicine colchicine hinders the formation of AAA in animal models 69, 70. As a potent anti-inflammatory medicine, colchicine primarily inhibits the infiltration of immune cells into the aortic wall, thereby reducing inflammation, oxidative stress, and pyroptosis, and preserves the contractile phenotype of VSMCs to further maintain vascular homeostasis. *Salvia miltiorrhiza Burge*, also known as Danshen, an eminent medicinal herb that exhibits potent anti-inflammatory activity, has been widely used in Asian nations to treat cardiovascular disorders such as myocardial infarction, atherosclerosis, and coronary heart diseases 71. Of note, both colchicine and CTS have been shown to be specific inhibitors of the NLRP3 inflammasome 72, 73. On the basis of the findings from this study, we deduced that* Salvia miltiorrhiza* was also a potential therapeutic agent for AAA. We and others have previously shown that CTS and the other three major tanshinones, dihydrotanshinone, tanshinone I, and tanshinone IIA, derived from *Salvia miltiorrhiza*, have actions in activating the Nrf2 pathway in human bronchial epithelial cells, human skin cells, and human macrophages 74-76. In the present study, we compared the activation capacity of Nrf2 among the four tanshinones, and our data showed that CTS exhibited the most robust induction of Nrf2 and one of its most important targets, HO-1, far more than other compounds detected in both RAVSMCs and MAVSMCs Figure S4C). Furthermore, compared to tanshinone IIA, CTS seems to have superior anti-inflammatory properties, as we previously observed (18. Compared to the chemical structure of the other three tanshinones, CTS stands out with its distinctive dihydrofuran ring at the C-15 position.

This unique feature likely enhances the anti-inflammatory properties of CTS and its capacity to strongly activate the Keap1-Nrf2-HO-1 pathway. Regarding the exact mechanism of Nrf2 activation by CTS, we identified the site of Arg415 located in Keap1 as one of the most vital competitive binding sites between Keap1 and Nrf2, as previously reported 66 through the combination use of virtual docking, site-directed mutagenesis, CETSA, and ITC technologies. The interaction between Arg415 and the Glu79 residue in the Neh2 domain of the Nrf2 protein, which is highly conserved, acts as a negative regulatory domain for Nrf2 transcriptional activity. The R415K mutation decreased the binding strength of the Neh2 peptide by almost tenfold 67, 77. When this site is occupied by CTS, it effectively hinders the binding of Keap1 to Nrf2, resulting in the degradation blockade and accumulation of Nrf2, and subsequent translocation to the nucleus, where it mediates the transcription of its target genes (e.g., *HMOX-1* and* NQO1*). A recent study demonstrated that echinacoside, another natural compound, upregulated Nrf2 protein expression by promoting Nrf2 acetylation 78. Further investigation is required to determine whether Nrf2 acetylation is involved in CTS-mediated Nrf2 expression upregulation.

A salient finding in this study is that CTS is capable of inhibiting pyroptosis to prevent AAA. Pyroptosis, as a form of controlled cell death, can be triggered by components of pathogens and signaling molecules from the host. Importantly, pyroptosis releases cytokines to facilitate the inflammatory response. Inhibiting cell death and inflammation in a formed vicious circle is likely to attenuate AAA by improving the inflammatory microenvironment of the abdominal aorta. Indeed, there is growing evidence that VSMC pyroptosis plays a crucial role in AAA formation and development 10-12. Current pyroptosis inhibitors are primarily in the preclinical stage, with the most favorable targets being NLRP3 and caspase 1 9. A previous study has shown that CTS was a specific inhibitor of the NLRP3 inflammasome in canonical and noncanonical manners and was capable of alleviating inflammatory diseases, such as endotoxemia syndrome, but still lacks direct evidence that CTS is able to inhibit pyroptosis 73. Our data demonstrated, for the first time, that CTS treatment effectively suppressed the initiation of VSMC pyroptosis in both *in vitro* and *in vivo*, as evidenced by the inactivation of NLRP3, inhibition of cleavage of caspase 1 and GSDMD, decreased release of IL-1β and IL-18, and blocking of pore formation on the cell membrane. VSMCs are of significant importance in the pathogenesis of aneurysms, but the involvement of other cell types should not be overlooked. In addition to VSMC pyroptosis, accumulation of macrophages in the aneurysmal aortic wall is one of the main pathological characteristics of AAA 79, which is also observed in our study (Figs. 1J and 9A). More recently, macrophage-derived pyroptosis has also been shown to contribute to AAA formation and development 80. Purinergic receptor P2X7 attenuated aneurysm formation in experimental murine AAA models by inhibiting the activation of the NLRP3 inflammasome, thus inhibiting the cleavage of downstream caspase-1 and the initiation of the pyroptosis pathway 81. Nevertheless, we have compared the induction of HO-1, a key Nrf2 target protein with anti-inflammatory, antioxidant, and anti-AAA properties 61, by CTS in VSMCs, ECs, and macrophages, with results indicating that VSMCs exhibited the strongest induction of HO-1 Figure S3D). More importantly, the therapeutic effect of CTS on AAA and the inhibitory effect of CTS on VSMC pyroptosis were largely abrogated by VSMC-specific *Nfe2l2* knockdown in mice, indicating the critical role of VMSCs in CTS-mediated protective effects in the process.

Similar to NLRP3, CTS is also a specific inhibitor of STAT3, which has been shown to be overactivated in AAA (82, 83. Additionally, we previously reported that CTS was able to suppress the activation of redox-sensitive transcription factor NF-κB via inhibition of LOX-1-mediated ROS production 18. Indeed, our findings showed that CTS inhibited TNF-α-induced phosphorylation of NF-κB-p65 and STAT3 in VSMCs Figure S7. Thus, it is possible that CTS attenuated AAA formation at least partly by inhibiting the activation of NF-κB and STAT3. Of note, our network pharmacology study, RNA-seq analysis and Nrf2 intervene experiments indicate that the Keap1-Nrf2 pathway is the predominant enriched pathway associated with the protective effects of CTS on AAA. In fact, a Nrf2-ROS-NF-κB pathway 84, 85, a Nrf2-NF-κB/STAT3 pathway 86, 87 and a NF-κB-pyroptosis pathway 88, 89 have been well established previously. Collectively, combined with our findings and others, it can be concluded that Nrf2 activation plays a central role in CTS-mediated inhibition of NF-κB/STAT3 signaling, inflammatory response, pyroptosis and AAA formation.

Despite its beneficial biological effects, CTS has minimal oral bioavailability due to its very limited water solubility. Therefore, a series of effective methods have been applied to increase the oral bioavailability of CTS. One of the methods employed in this study is cyclodextrin inclusion, which has a hydrophilic outer surface and a lipophilic central cavity, to enhance solubility after forming complexes with compounds 21. Moreover, decreasing the particle size of CTS through micronization to enhance the surface area and accelerate dissolution is another efficient method 90. Using solid lipid nanoparticles, made of lipid components that are well-tolerated in the body, as carriers of CTS delivery is also a useful strategy due to their reduced particle size, similar to micronization, and their ability to prevent drug degradation by lipid 91. Recently, our laboratory also managed to load CTS into macrophage-targeted liposomes and fused with microparticles using spray drying for effective pulmonary administration to treat pulmonary fibrosis 92. Future studies on the characteristics of CTS will be beneficial to investigate more effective drug delivery strategies to further improve the oral bioavailability of CTS. Apart from this, a more direct path is to modify the chemical structure of CTS to change its properties. Over 30% of medications approved by the U.S. FDA are either natural compounds or derived directly from natural substances. LYW-6, a novel derivative of CTS, presents better affinity and inhibition capacity for transcription factor STAT3 than CTS in the treatment of colorectal cancer, with much reduced toxicity 93. Two metabolites of CTS produced by the fungus Mucor rouxii AS 3.3447 exhibit improved anti-influenza A virus properties and lower toxicity due to the breakdown and rearrangement of the ortho-naphthoquinone chromophore of CTS 94. Therefore, altering the structure of CTS to increase its oral bioavailability and anti-AAA activity is another important focus of our next step.

## Conclusions

In this work, we describe for the first time the protective effect of CTS against AAA and provide some insight into the precise mechanism by which CTS inhibits AAA. CTS protects against AAA by suppressing oxidative stress, inflammatory response, and pyroptosis while maintaining ECM equilibrium and the VSMC contractile phenotype via activating the Keap1-Nrf2 signaling axis through competitive binding to Keap1 at Arg415. Our study reveals that CTS may be developed as a promising pharmacological therapy for the treatment of AAA.

## Supplementary Material

Supplementary figures and tables.

## Abbreviations

AAA: abdominal aortic aneurysm
AAV: adeno-associated virus
Ang II: angiotensin II
ApoE^-/-^: apolipoprotein E knock-out
Arg: arginine
CCK-8: cell counting kit-8
Ccl2: monocyte chemoattractant protein-1
CD68: cluster of differentiation 68
CETSA: cellular thermal shift assay
Cle-Caspase 1: cleaved-caspase 1
CTS: cryptotanshinone
DEGs: differentially expressed genes
DHE: dihydroethidium probe
DO: disease ontology
DOXY: doxycycline
ECAR: extracellular acidification rate
ECM: extracellular matrix
EVG: Verhoeff-Van Gieson elastic staining
FBS: fetal bovine serum
GO: gene ontology
GSDMD: gasdermin D
H&E: hematoxylin & eosin
H_2_O_2_: hydrogen peroxide
HBSS: Hank's balanced salt solution
HO-1: heme oxygenase 1
HP-β-CD: hydroxypropyl-beta-cyclodextrin
IHC: immunohistochemistry
IL-1β: interleukin 1 beta
IL-18: interleukin 18
ITC: isothermal titration calorimetry
ITDRF: isothermal dose-response fingerprint
Keap1: kelch-like ECH-associated protein 1
KEGG: Kyoto Encyclopedia of Genes and Genomes
LDH: lactate dehydrogenase
LOX-1: lectin-like ox-LDL receptor-1
Lys: lysine
MAVSMCs: mouse aortic VSMCs
MDA: malondialdehyde
MMPs: matrix metalloproteinases
mtDNA: mitochondrial DNA
NLRP3: NOD-like receptor thermal protein domain associated protein 3
Nrf2: nuclear factor erythroid 2-related factor 2
OCR: oxygen consumption rate
qRT-PCR: quantitative real-time PCR
RAVSMCs: rat aortic VSMCs
SD: Sprague-Dawley
RNA-seq: RNA sequencing
SEM: scanning electron microscope
SM22α: smooth muscle 22α
SOD: superoxide dismutase
TBHQ: tertiary butylhydroquinone
TEM: transmission electron microscopy
TNF-α: tumor necrosis factor-α
VCAM-1: vascular cell adhesion molecule 1
VSMCs: vascular smooth muscle cells
α-SMA: α-smooth muscle actin
18S rRNA: 18S ribosomal RNA
