# Supplementary material for: Targeting the smooth muscle cell Keap1-Nrf2-GSDMD-pyroptosis axis by cryptotanshinone prevents abdominal aortic aneurysm formation
Source: Theranostics. 2024 Oct 7;14(17):6516–42. doi: 10.7150/thno.98400 (PMC11519792; doi:10.7150/thno.98400)
Supplement: Supplementary file 1 — Supplementary figures and tables. [file thnov14p6516s1.pdf]

Supplementary material

Supplementary figures

A B

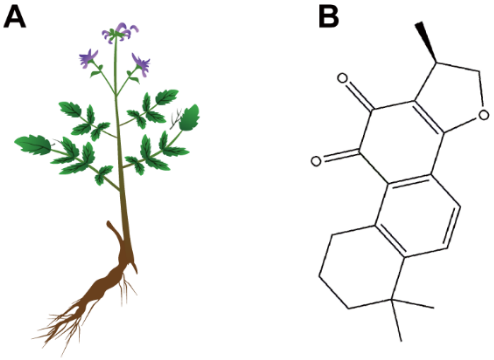

Figure S1. (A-B) *Salvia miltiorrhiza Bunge* (A) and the chemical structure of cryptotanshinone (B).

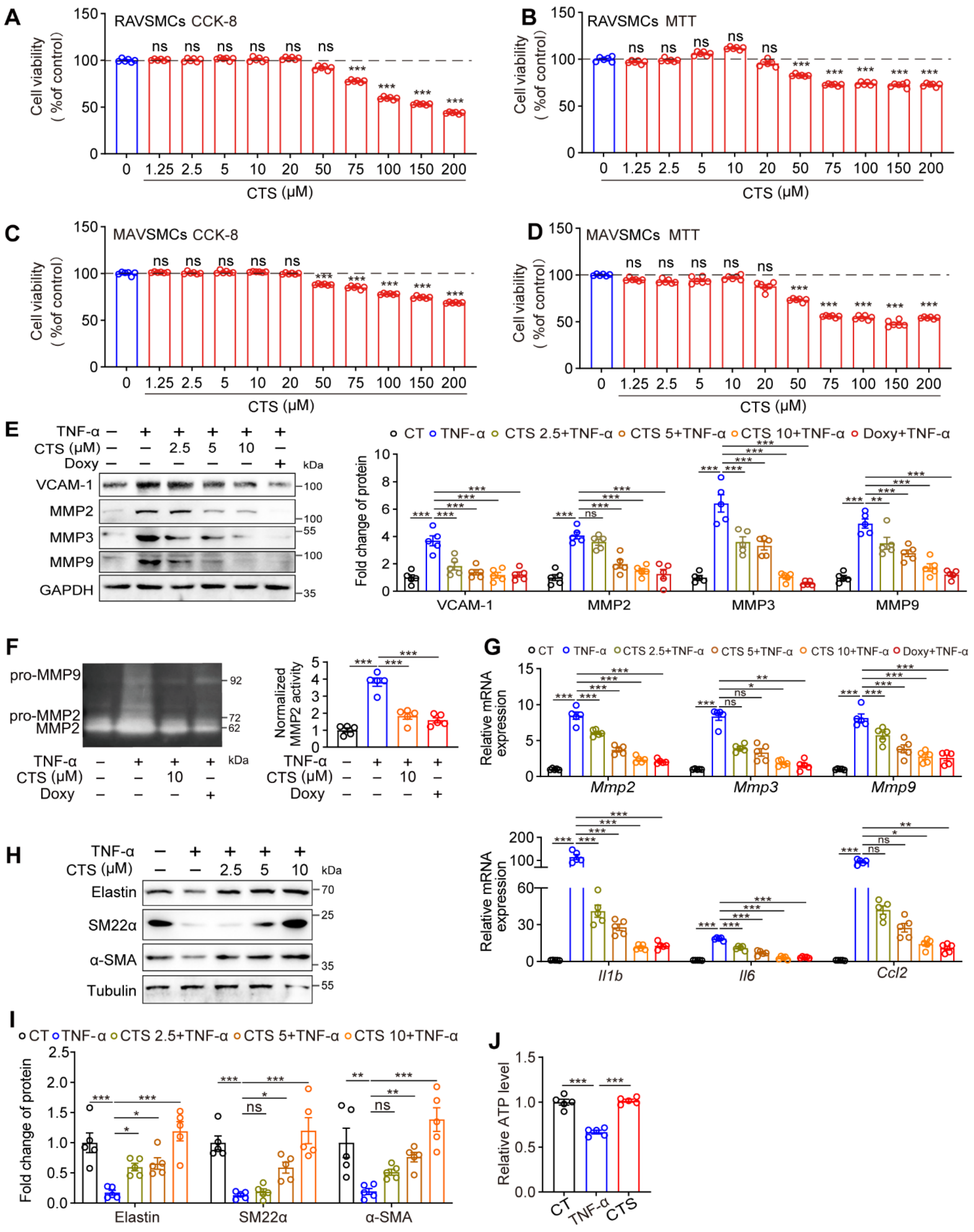

Figure S2. (A-D) CCK-8 and MTT assays were performed to check cell viability on RAVSMCs and MAVSMCs used in this study at indicated doses of CTS for 24 hours. **(E)** Representative western blots and quantitative analysis of the indicated protein expression in MAVSMCs treated with TNF-α, CTS, and doxycycline (n = 5). **(F)**

Representative gelatin zymogram images and enzymatic activity analysis of MMP2 and MMP9 (n = 5). (**G**) qRT-PCR analysis of the mRNA expression of MMPs and cytokines in RAVSMCs treated with TNF-α, CTS, and doxycycline (n = 5). **(H-I)** Representative western blots and quantitative analysis of the indicated protein expression in MAVSMCs treated with TNF-α and CTS. (**J**) ATP levels were measured from the cell culture supernatants of the indicated treatments in RAVSMCs (n = 5). Data are presented as mean ± SEM. $^{*}p < 0.05$, $^{**}p < 0.01$, $^{***}p < 0.001$, ns: no significant.

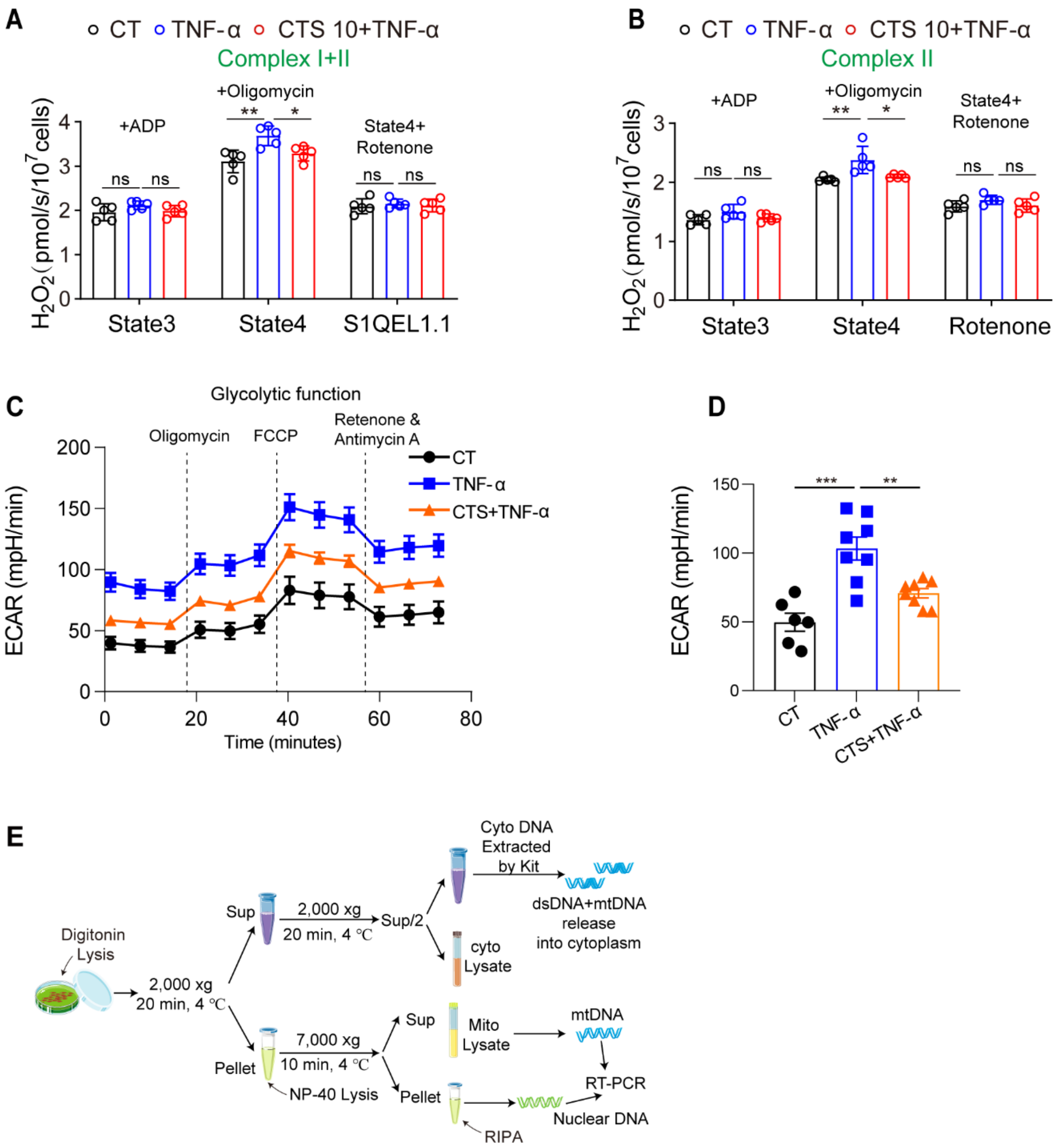

Figure S3. (**A-B**) H_2O_2 production was measured from the RAVSMCs (n = 5). (**C-D**) ECAR profile showing glycolytic function in RAVSMCs. (D) Quantification of glycolytic function parameters from A. n = 6-8. (**E**) Workflow of the cytosolic dsDNA and mtDNA extraction processes.

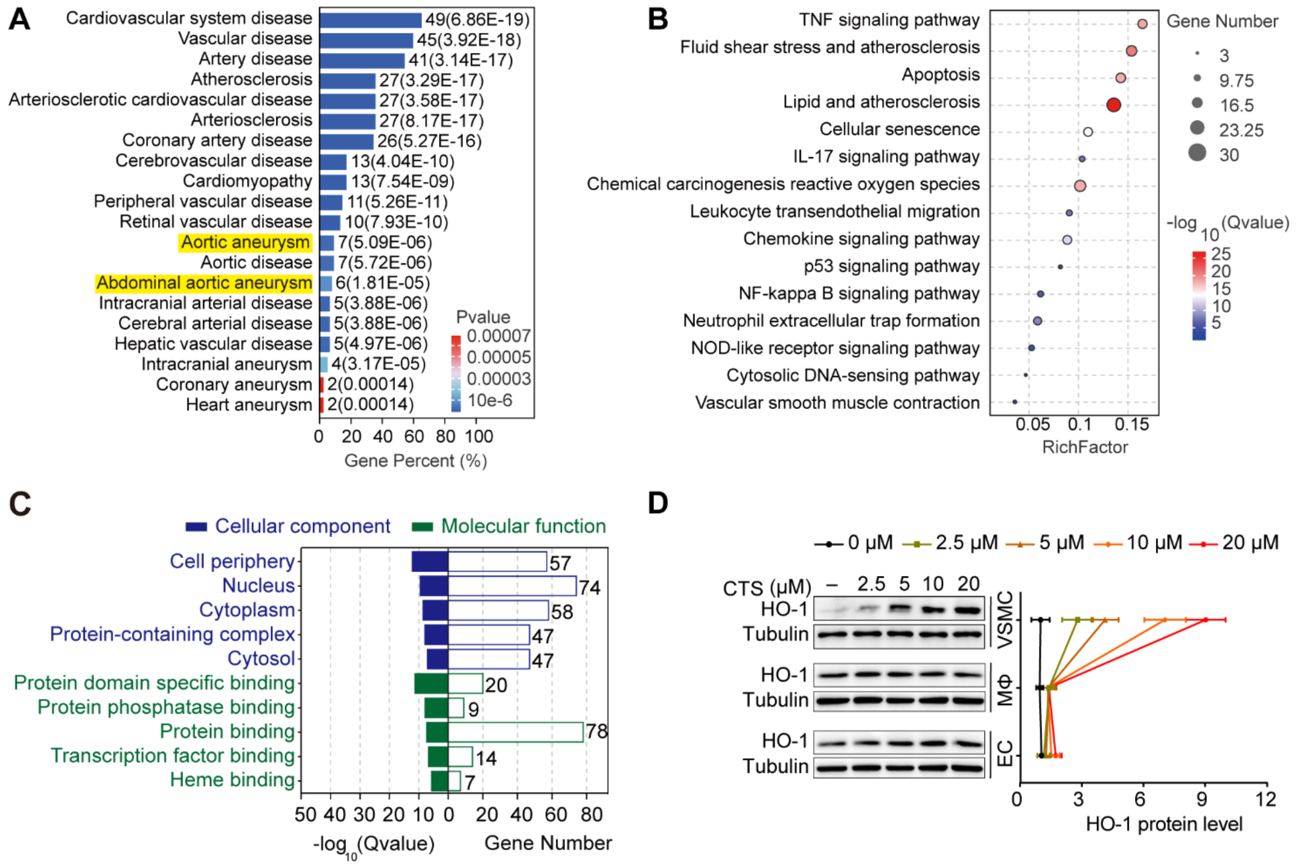

Figure S4. (A) The DO enrichment analysis revealed that the 192 common genes were primarily associated with vascular diseases. In this analysis, the color and length of the bands were utilized to signify the *P*-value and the percentage of genes. **(B)** KEGG analysis of the intersection targets of AAA and CTS based on network pharmacology. In the KEGG analysis, the color and dimension of the bubbles represented the *P*-value and the gene count. **(C)** The bidirectional bar chart presents the results of enrichment analyses in the cellular component (blue bars) and molecular function (green bars) categories post-GO analysis. Colored bars quantify the significance of common-target enrichment as log10 (Q values), with longer bars signifying higher levels of statistical significance. Conversely, the unfilled bars correspond to the count of common-targeted genes associated with each category, offering a quantitative insight into the gene distribution pattern. **(D)** Representative western blots and quantitative analyses of HO-1 protein levels in VSMCs, macrophages (MΦ) and ECs. RAVSMCs were pre-treated with various concentrations of CTS. Cells were harvested for western blot at 24 hours (n = 3).

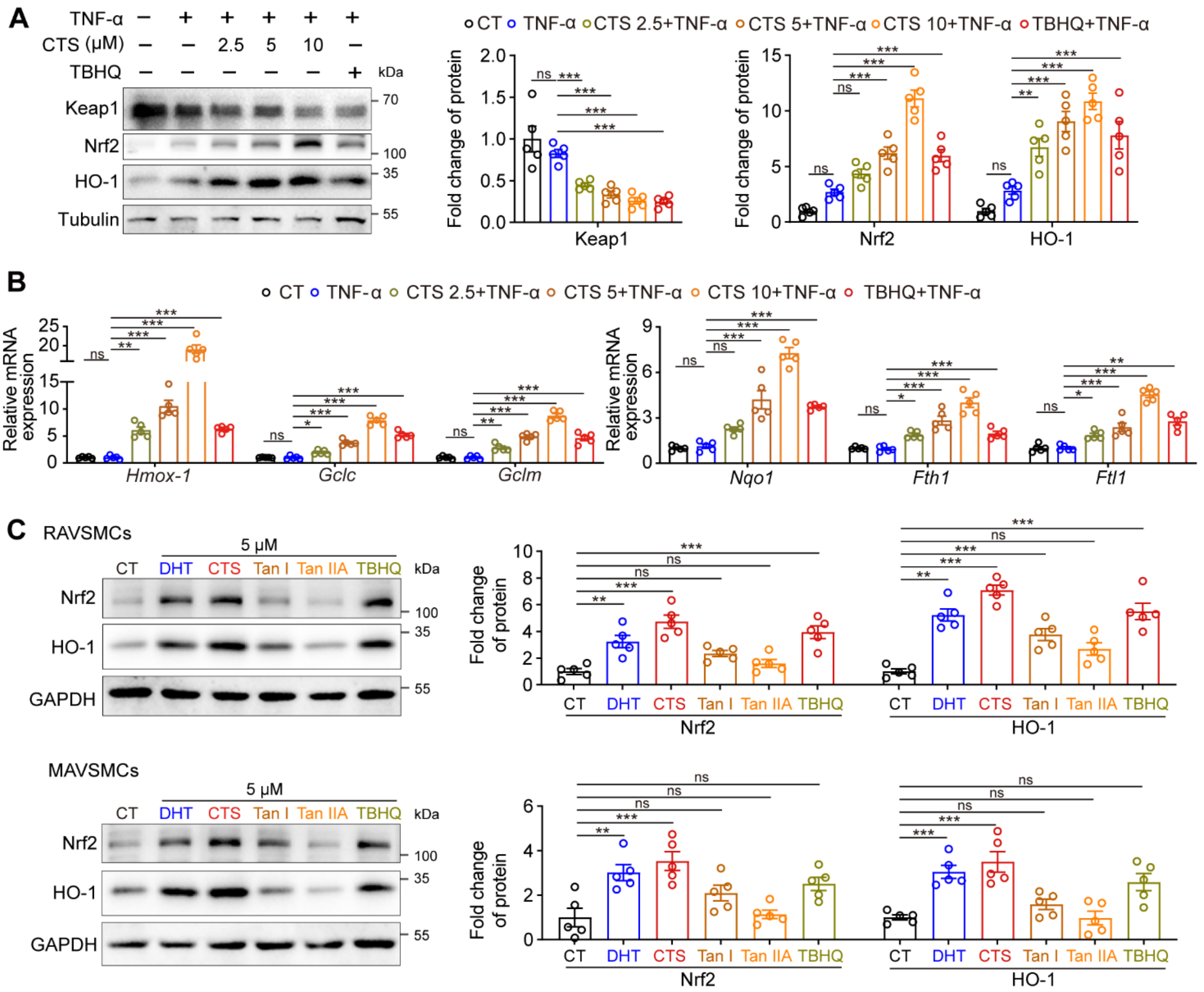

Figure S5. (A) Representative western blots and quantitative analyses of the indicated protein expressions in MAVSMCs treated with different doses of CTS (2.5, 5, and 10 μM). TBHQ is an activator of the Nrf2 pathway used as a positive control (n = 5). (**B**) qRT-PCR analysis of the mRNA expression of Nrf2 target genes in RAVSMCs treated with TNF-α, CTS, and TBHQ (n = 5). **(C)** RAVSMCs and MAVSMCs were subjected to treatment with various concentrations of other tanshinone monomers. Following a 24-hour incubation period, Nrf2 and HO-1 protein levels were assessed by Western blot analysis across the varied doses of the distinct monomers (n = 5). Data are presented as mean ± SEM. *$p < 0.05$, **$p < 0.01$, ***$p < 0.001$, ns: no significant.

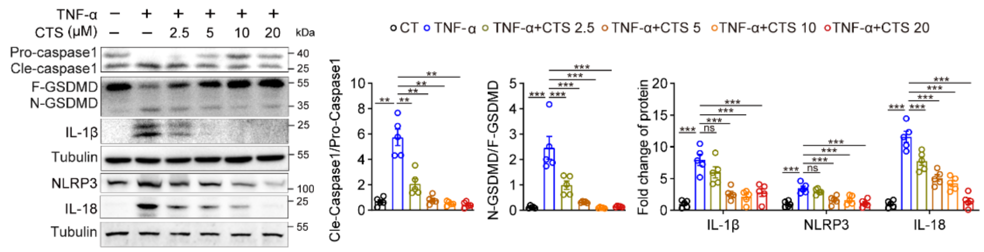

Figure S6. Representative western blots and quantitative analysis of the indicated protein expression in MAVSMCs treated with TNF-α and CTS (n = 5). Data are presented as mean ± SEM. *p < 0.05, **p < 0.01, ***p < 0.001, ns: no significant.

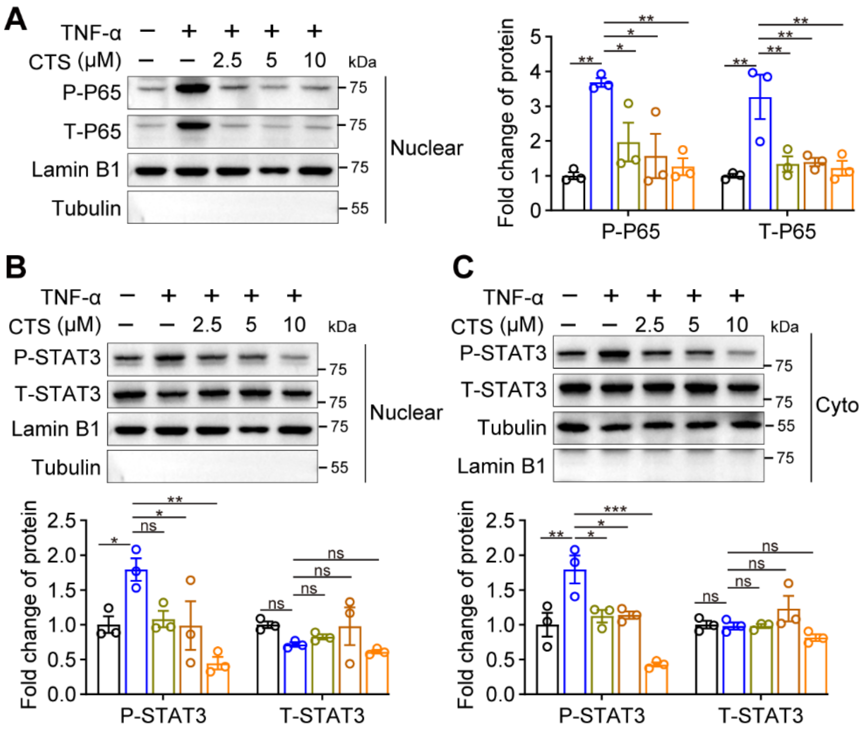

Figure S7. Representative western blots and quantitative analysis of the indicated protein expression in RAVSMCs treated with TNF-α and CTS (n = 3). Data are presented as mean ± SEM. *p < 0.05, **p < 0.01, ***p < 0.001, ns: no significant.

Supplementary tables

Table S1: Antibodies

Antibody	Source	Catalog No.	Dilution
Caspase-1	Proteintech	22915-1-AP	1:2,000 for WB<br>1:200 for IHC
CD68	Proteintech	28058-1-AP	1:400 for IHC
Elastin	Signalway Antibody	45078	1:1,000 for WB
GAPDH	Proteintech	60004-1-Ig	1:20,000 for WB
HO-1	Proteintech	10701-1-AP	1:2,000 for WB<br>1:200 for IHC
IL-18	Abclonal Technology	A1115	1:1,000 for WB
IL-1β	Proteintech	26048-1-AP	1:1,000 for WB
Keap1	ZENBIO	R26935	1:1,000 for WB
Lamin B1	ZENBIO	R24825	1:1,000 for WB
MMP2	Santa Cruz Biotechnology	sc-13595	1:500 for WB<br>1:200 for IHC
MMP3	Proteintech	66338-1-Ig	1:5,000 for WB<br>1:200 for IHC
MMP9	Abcam	ab38898	1:1,000 for WB<br>1:200 for IHC
GSDMD (Full length+N terminal)	Abclonal Technology	A20197	1:1,000 for WB<br>1:200 for IHC
NLRP3	Cell Signaling Technology	15101	1:1,000 for WB<br>1:200 for IHC
NQO1	Santa Cruz Biotechnology	sc-32793	1:1,000 for WB
Nrf2	Proteintech	16396-1-AP	1:1,000 for WB<br>1:200 for IHC<br>1:100 for IF
SM22α	GeneTex	GTX628831	1:2,000 for WB<br>1:200 for IHC
Tubulin	Proteintech	11224-1-AP	1:10,000 for WB
VCAM-1	ZENBIO	383318	1:1,000 for WB<br>1:200 for IHC
α-SMA	Santa Cruz Biotechnology	sc-56499	1:500 for WB<br>1:200 for IHC

Table S2: Primers

Gene	Forward (5'-3')	Reverse (5'-3')
Rat *Ccl2*	ggtctctgtcacgcttctgg	gggcattaactgcatctgg
Rat *Fth1*	atcaaccgccagatcaacc	aagttcttcagggccacatc
Rat *Ftl1*	tctcaagatgagtggggtaaaa	gcgtgcagatccaagagg
Rat *Gapdh*	aggagtaagaaaccctggac	ctgggatggaattgtgag
Rat *Gclc*	aagcctcctcctccaaactc	cctggtcagcagtaccacaa
Rat *Gclm*	agcatttgcagccttactgg	gctgctccaactgtgttttg
Rat *Hmox1*	caggtgtccagggaaggctttaag	tgggttctgcttgtttcgctctatc
Rat *Il1b*	gtcactcattgtggctgtgg	agacagcacgaggcattttt
Rat *Il6*	tcttggtccttagccactcc	tccttcctaccccaacttcc
Rat *Mmp2*	agtggatgatgcctttgctc	tgggtatccatctccatgct
Rat *Mmp3*	acccaaatggaggaaaaacc	tctcaatggcagaatccaca
Rat *Mmp9*	caccgccaactatgaccaggataag	ctgcttgcccaggaagacgaag
Rat *β-actin*	gctgtgctatgttgccctagacttc	ggaaccgctcattgccgatagtg
Mouse *Gapdh*	aggtcggtgtgaacggatttg	ggggtcgttgatggcaaca
Mouse *18s rRNA*	tcaagaacgaaagtcggagg	ggacatctaagggcatcac
Mouse mt-*Atp6*	acaccaaaaggacgaaca	aggaagtgggcaagtgag
Mouse mt-*Cox I*	tcggagccccagatatagca	tttccggctagaggtgggta
Mouse mt-*Cox II*	taatagacgaaatcaacaacc	caaagcataggtcttcatagt
Mouse mt-*Nd1*	cgacctgacagaaggaga	gtaacggaagcgtggata
Keap1 mutation primer	agcgtgccccgtaacAAGatcggggtgggg	gcgggggtactcgcacggggcattgTTC
